# On the robustness of Bayesian inference of gene flow to intragenic recombination and natural selection

**DOI:** 10.1093/molbev/msaf327

**Published:** 2025-12-12

**Authors:** Yuttapong Thawornwattana, Bruce Rannala, Ziheng Yang

**Affiliations:** Department of Genetics, Evolution, and Environment, University College London, Gower Street, London WC1E 6BT, UK; Department of Evolution and Ecology, University of California, Davis, CA 95616, USA; Department of Genetics, Evolution, and Environment, University College London, Gower Street, London WC1E 6BT, UK

**Keywords:** Bpp, introgression, migration, multispecies coalescent, recombination, selection

## Abstract

The multispecies coalescent (MSC) model provides a framework for detecting gene flow using genomic data, including between sister species. However, the robustness of the inference to violations of model assumptions are poorly understood. Here, we use simulation to study the false positive rate of a Bayesian test of gene flow under the MSC with multiple influencing factors including recombination, natural selection, discrete versus continuous gene flow, variable species divergence time, and gene flow involving sister versus nonsister lineages. We find that in almost all scenarios examined the test has very low false positives. However, the test of gene flow between sister lineages may be prone to high false positives in cases of very recent species divergence and very high recombination rate. At low recombination rates, the test is robust to selective sweeps, background selection and balancing selection, although prolonged balancing selection can lead to false signals of gene flow between sister lineages. The impact of excessive recombination on the test of gene flow between sisters may be assessed by using a smaller number of sequences for each species and by considering shorter sequences at each locus. Recent species divergence alone (with no recombination) does not cause false positives in tests of gene flow, contrary to previous claims. The test of gene flow between nonsister lineages is robust to recombination at all divergence levels. Our findings provide guidance for reliable inference of gene flow using coalescent methods and highlight the need for care in conducting and interpreting simulation experiments.

## Introduction

With recent advances in methodological and software development (Jiao et al. 2021; Mirarab et al. 2021; Hibbins and Hahn 2022) and increased availability of genomic data, the multispecies coalescent (MSC) provides a powerful statistical framework for inferring historical demography and gene flow from genomic data. The MSC method makes several modeling assumptions, including no intra-locus recombination, high inter-locus recombination, neutral evolution, and random mating (no population structure) (Jiao et al. 2021). Although the MSC could be extended to model recombination within population, this is a serious theoretical and computation challenge and existing implementations of the MSC model therefore do not explicitly account for recombination. When the MSC model is applied to multi-locus sequence data, a single genealogical tree is assumed to apply to all sites at a locus while different loci are assumed to have independent histories (Nielsen and Wakeley 2001; Rannala and Yang 2003). Intra-locus recombination is expected to be a more serious model violation than tightly linked loci, because gene tree correlations among loci due to linkage should influence the information content in the data only (similar to the use of a composite likelihood), whereas intra-locus recombination causes a violation of the likelihood model. Several simulation studies suggest that inference under the MSC model is largely robust to realistic levels of intra-locus recombination (Lanier and Knowles 2012; Zhu et al. 2022; Yan et al. 2023). The population recombination rate (ρ=4Nr per site, where *N* is the effective population size and *r* is the recombination rate per site per generation) used in these studies is up to 0.02 (Lanier and Knowles 2012), 0.005 (Zhu et al. 2022) and 0.008 (Yan et al. 2023). These values are much larger than the genome average for humans of about 0.0005 (Reich et al. 2002; Jensen-Seaman et al. 2004). However, *ρ* in organisms such as insects can be orders of magnitude larger, due to more frequent recombination per site per generation (*r*) and larger effective population sizes (*N*) (DeLory et al. 2024). For example, in *Drosophila melanogaster*, *ρ* will be about 0.1, if $r=2.3\times 10^{-8}$ per site per generation (Singh et al. 2005) and $N=10^{6}$. The impact of such high recombination rates on inference under the MSC has not been explored. Moreover, the impact of recombination in combination with other factors such as species divergence time, the mode of gene flow, and selection remains largely unknown.

Current implementations of the MSC model also assume neutral sequence evolution. Natural selection may alter the pattern of shared polymorphism within and between species, potentially creating false signals of gene flow (Cruickshank and Hahn 2014; Smith and Hahn 2024). For instance, loci under balancing selection (frequency-dependent selection) that originated in the ancestral population and have persisted to present-day populations can mimic a signal of gene flow due to shared ancestral polymorphism. Background selection tends to reduce polymorphism at linked neutral loci across the genome (Charlesworth et al. 1993; Hudson and Kaplan 1995; Nordborg et al. 1996). Thus its effect on the MSC inference should be primarily a reduction in estimates of the effective population size and species divergence times (Shi and Yang 2018). Selective sweeps can also lead to reduced polymorphism around selected sites (Maynard Smith and Haigh 1974). However, selective sweeps may not have a large impact on inference under the MSC because beneficial mutations should be rarer than deleterious ones and their effects are transient (Przeworski 2002). Indeed in simulations MSC-based inference was found to be robust to positive selection (Adams et al. 2018; Wascher and Kubatko 2023).

In contrast to the findings outlined above a recent study by Smith and Hahn (2024) found that different types of natural selection caused excessive false positives when data were simulated under different schemes of selection without gene flow and analyzed using three methods to detect introgression between sister populations: Bpp (Yang 2015; Flouri et al. 2018, 2020), ∂a∂i (Gutenkunst et al. 2009), and Fastsimcoal2 (Excoffier et al. 2013). Bpp is a full-likelihood method under the MSC applied to multilocus sequence data, whereas ∂a∂i and Fastsimcoal2 use a composite likelihood of site frequency spectrum (SFS) data assuming independent sites. The authors concluded that by ignoring selection, all three methods generate false-positive signals of introgression in certain parameter regimes. However, the study design is flawed and their interpretations of the simulation results are misleading (see Discussion). The authors considered a Bayesian test to be significant if the 95% highest posterior density (HPD) credibility interval (CI) for the introgression probability excludes zero, an approach that is sensitive to the prior on parameters. While the authors ascribed the false positives to selection, their results showed an overwhelming effect of the species split time, whereas differences among the selection schemes were much less important (Smith and Hahn 2024, Figs. 1, 3, and 5 for ∂a∂i , Fastsimcoal2, and Bpp, respectively). In particular, Bpp had 100% false positive rates at a shallow divergence time (T=N generations) regardless of the selection scheme (including neutral evolution) whereas the false positive rates were low at deeper divergence (T=4×N and 16×N). As a result it is unclear what may have caused the high false positives reported by Smith and Hahn (2024).

Gene flow between sister populations is expected to be harder to detect than between nonsisters (Hibbins and Hahn 2022). For example, gene flow between nonsisters may cause asymmetries in the gene tree distribution and in site-pattern counts in genomic data and can thus be detected by summary quartet methods such as *D* (or Hyde) (Green et al. 2010; Blischak et al. 2018) and Snaq (Solis-Lemus and Ane 2016). In contrast, gene flow between sisters is undetectable by those methods. Methods that detect gene flow between sister species typically use multiple samples from the same species and use more information than the gene-tree frequencies or genome-wide site-pattern counts. However, Cruickshank and Hahn (2014, Fig. 4) found that inference of gene flow between two species using the Ima2 program (Hey 2010) generated excessive false positives at very shallow divergence, in particular in small datasets with a few loci. This was confirmed by Hey et al. (2015) but no practical solution has been proposed. The high false positives are surprising because in those studies, data were simulated assuming no recombination and no selection, and all assumptions of the MSC model were satisfied.

Recent research also suggests possible differences in the test of gene flow conducted under the discrete MSC-introgression (MSC-I) model versus the continuous MSC-migration (MSC-M) model (Huang et al. 2022; Thawornwattana et al. 2025; Yang et al. 2025). For example, when the rate of gene flow between sisters is very low, the estimated migration rate under MSC-M is close to 0 (Thawornwattana et al. 2025, Fig. 3), whereas the estimated introgression probability under MSC-I may not be close to 0, but rather fluctuate over the whole range (0,1) (Huang et al. 2022, Fig. 3). Those results hint at possible differences depending on whether the MSC-I or MSC-M model is used to conduct the test. Indeed the test of introgression between sisters under MSC-I has a number of “nonstandard conditions” (e.g. Self and Liang 1987, Fig. 3), such as boundary problems, unidentifiability issues, and multiple routes from the alternative to the null hypotheses (Yang et al. 2025). In comparison, the corresponding test under the MSC-M model appears not to involve most of those irregularities.

**Fig. 1.**
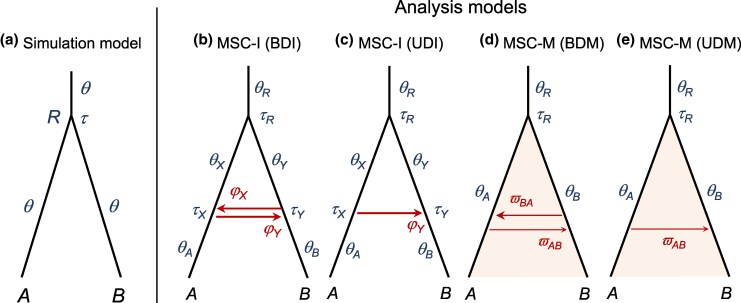
a) Simulation and b)–e) analysis models. Data are simulated under the MSC model with no gene flow a) and analyzed under the discrete introgression model (MSC-I, b&c) or the continuous migration model (MSC-M, d&e) with either bidirectional (b&d) or unidirectional (c&e) gene flow, to calculate the Bayes factor to conduct the Bayesian test.

Here, we conduct an extended simulation to examine the false positive rate of a Bayesian test of gene flow, influenced by several aspects of the data and model. We consider a variety of recombination rates, different schemes of natural selection, the size and shape of the species phylogeny, the species divergence time, the size of the dataset, the mode of gene flow (MSC-I versus MSC-M) with either unidirectional or bidirectional gene flow (Fig. 1b–e), and gene flow involving either sister or nonsister populations. Smith and Hahn (2024) used the bidirectional introgression model (Fig. 1b) only. Instead of the HPD CI used in the introgression test of Smith and Hahn (2024), we apply the Bayes factor to conduct the Bayesian test of gene flow, which is the standard device for hypothesis testing in the Bayesian framework (Jeffreys 1935).

We find that excessive false positives for the Bayesian test of introgression reported by Smith and Hahn (2024) are due to a combination of very high recombination rate and very shallow species divergence, rather than natural selection as the authors claimed. We characterize the performance of the Bayesian test in multiple settings of parameters and data and in particular assess the effects of recombination, selection, and species divergence time. Overall the Bayesian test appears to be highly robust to recombination and selection. Even with excessive recombination, false positives are rare at high divergences between sister species and at all species divergences between nonsisters. We demonstrate that the high false positives for the likelihood ratio test (LRT) of gene flow reported by Cruickshank and Hahn (2014) and Hey et al. (2015) are due to inappropriate use of integrated likelihood and misinformative uniform priors in the Ima program. We show that the so-called “small data-low divergence” (SDLD) problem (Hey et al. 2015) is not a problem for a conventional Bayesian test using the Bayes factor. Our results should serve as useful guides for applying coalescent-based tests of gene flow using genomic data.

## Results and discussion

Here, we summarize the results of simulations examining the false positive rate of the Bayesian test of gene flow under both the MSC-I and MSC-M models. We simulated data under the null model of no gene flow, with no or variable amounts of recombination and with neutral evolution or several modes and intensities of natural selection (see the summary in Table S1 and the Materials and Methods section). We also consider a range of population divergence times and data sizes (in the number of sequences sampled per population). Our main focus in this study is the robustness of the Bayesian test of gene flow in the MSC modeling framework. In all cases, the Bayes factor $B_{10}$ measures the evidence in support of the alternative hypothesis $H_{1}$ of gene flow (either continuous migration or discrete introgression) against the null hypothesis $H_{0}$ of no gene flow. Since we are concerned with false positives rather than the power of the test to detect gene flow, all datasets are simulated under the null model (with no migration or introgression) but other factors such as selection and recombination are varied among simulations. We examined biases in estimates of parameters as well (including rates of gene flow), but note that the true rates are zero in datasets simulated in this study.

### Performance of Bayesian test: no recombination or selection

In our standard simulation, we evaluated the false positive rate of the Bayesian test of gene flow between sister species when the data are generated with either intralocus recombination or selection but no gene flow. We simulated data using the phylogeny for two species of Fig. 1a, with three species divergence times, at T=N, 4N and 16N generations (or τ=θ/4, *θ* and 4θ), and then analyzed them under both the discrete MSC-I model and the continuous MSC-M model, assuming either unidirectional or bidirectional gene flow (BDI, UDI, BDM, and UDM, Fig. 1b–e). Under the MSC-I models (BDI and UDI), we also considered model specifications that placed constraints on population sizes (*θ*) on the phylogeny, reducing the number of parameters. As the data were simulated with no gene flow, any inferred gene flow will be a false positive.

**Fig. 2.**
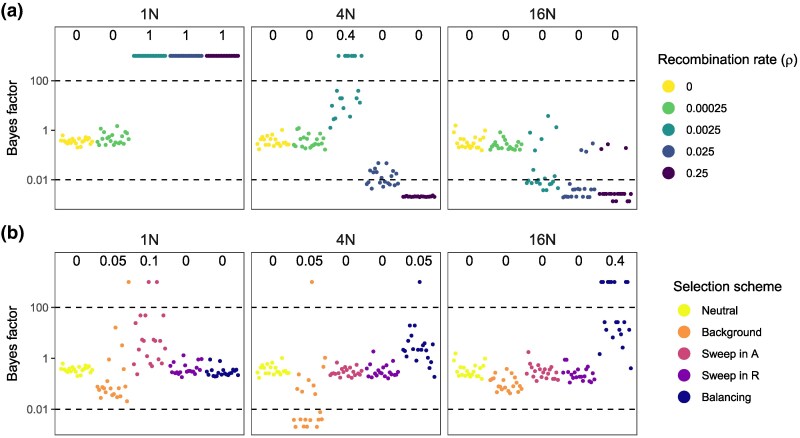
Bayes factors ($B_{10}$) for testing gene flow under the BDI model in analysis of 20 replicate datasets simulated under a) neutral evolution with different recombination rates and b) under different selection schemes without recombination. $B_{10}$ is calculated using the Savage-Dickey density ratio with ϵ=0.001 (Ji et al. 2023); those calculated using different ϵ as well as parameter estimates are shown in Figures S1 & S9. Columns correspond to three species divergence times: T=N,4N, 16N (or $\tau =\frac{1}{4}\theta ,\theta ,4\theta$). Horizontal dashed lines indicate the significance cutoffs at 100 and 0.01. Bayes factors larger than 1,000 are shown as 1,000. False positive rates are shown at the top of each subplot.

As a reference for comparison we first examine the behavior of the Bayesian test when there is no recombination or selection, i.e. when there is no model violation (Fig. 2a, ρ=0 or Fig. 2b, Neutral). Note that each of the four models of gene flow of Fig. 1b–e contains the null MSC model ($H_{0}$, Fig. 1a) as a special case and can be used as the alternative hypothesis ($H_{1}$) in the test. The asymptotic theory of Bayesian model comparison then predicts that when the number of loci L→∞, the null hypothesis $H_{0}$ dominates and the Bayes factor in support of the alternative hypothesis $H_{1}$, $B_{10}\to 0$ (e.g. Dawid 2011). With absent or uninformative data, $B_{10}=1$. In our simulation the test of gene flow using each of the four alternative hypotheses has false positive rates close to 0% (Figures S1–S8, ρ=0). We also note that in none of the datasets was $B_{10}<0.01$. At the shallow divergence, $B_{10}$ is around 1 in most datasets, although at the high divergence $B_{10}\approx 0.4$ in most datasets (Figures S1a, S2a, S3a, for ρ=0). There is more information about gene flow or its absence in datasets simulated at the high divergence, but overall the information content is low in datasets of this size (relative to the expectation for infinite data of $B_{10}\to 0$).

To interpret the parameter estimates, note that the test under the MSC-I models (BDI and UDI) involves several nonstandard features (Yang et al. 2025). For example the null MSC model $H_{0}$ does not correspond to one point in the parameter space of BDI ($H_{1}$) but instead is represented by five lines or planes (Table 1(i)). The posterior CIs for $\varphi_{X},\varphi_{Y}$ are very wide and cover nearly the whole range (0,1) (Figures S1b, S2b, S3b, for ρ=0). Furthermore, the BDI model without constraints on *θ*s is affected by an unidentifiability of the label-switching type, involving the four parameters $\varphi_{X},\varphi_{Y}$, $\theta_{X}$, and $\theta_{Y}$ (Yang and Flouri 2022). This type of unidentifiability occurs often in mixture models. Suppose there are two groups in proportions $p_{1},p_{2}=1-p_{1}$ and with means $\mu_{1},\mu_{2}$. Then the parameter vectors $\theta =(p_{1},\mu_{1},\mu_{2})$ and $\theta^{\prime }=(1-p_{1},\mu_{2},\mu_{1})$ are unidentifiable as they involve switching labels for groups 1 and 2. Models involving unidentifiability of the label-switching type can still be used in inference. The unidentifiability of the BDI model caused $\theta_{X},\theta_{Y}$ (as well as $\varphi_{X},\varphi_{Y}$) to have large CIs (Figure S1). With constraints on *θ*, this unidentifiability disappears so that $\theta_{X}=\theta_{A}$ and $\theta_{Y}=\theta_{B}$ are well estimated (even though $\varphi_{X},\varphi_{Y}$ are not) (Figures S2b & S3b  ρ=0).

**Table 1:** Parameters of interest and and indeterminate parameters in the Bayesian test under the BDI and UDI models (Yang et al. 2025)

Segment	Parameters of interest	Indeterminate
(**i**) Bidirectional introgression (BDI, Fig. 1b)		
(a)	$\varphi_{X}=0,\varphi_{Y}=0$	($\tau_{X}$)
(b)	$\varphi_{X}=1,\varphi_{Y}=1$	($\tau_{X}$)
(c)	$\beta =\frac{\tau_{X}}{\tau_{R}}=1$	($\varphi_{X},\varphi_{Y}$)
(d)	$\varphi_{X}=0,\varphi_{Y}=1$	($\tau_{R}$)
(e)	$\varphi_{X}=1,\varphi_{Y}=0$	($\tau_{R}$)
(**ii**) Unidirectional introgression (UDI, Fig. 1c)		
(a)	φ=0	($\tau_{X}$)
(b)	$\beta =\frac{\tau_{X}}{\tau_{R}}=1$	(φ)
(c)	φ=1	($\tau_{R}$)

Note. For BDI, segments (a), (b), (d), and (e) are lines, while segment (c) is a plane, and in segments (d) and (e), nuisance parameter $\tau_{X}$ in $H_{1}$ is mapped to *τ* in $H_{0}$ (Fig. 1a&b). For UDI, in segment (c) nuisance parameter $\tau_{X}$ in $H_{1}$ is mapped onto *τ* in $H_{0}$ (Fig. 1a&c).

The analysis under the UDI model with *A*-to-*B* introgression (Fig. 1c) is similar (Figures S4–S6, ρ=0). $H_{0}$ is represented by three lines or line segments in the parameter space of $H_{1}$ (Table 1(ii)). As a result, in data simulated with no gene flow, the posterior of $\varphi_{X\to Y}$ had wide CIs (Figures S4b, S5b, S6b, ρ=0). Again, irrespective of possible constraints on *θ*, $B_{10}$ tends to vary between 0.3-0.5 at the three divergence levels. In none of the datasets was $B_{10}<0.01$. The information content about $\varphi_{X\to Y}$ is relatively low, in sharp contrast with the very narrow CIs for some other parameters such as *θ* (Figure S6b, ρ=0).

The test under the MSC-M models (BDM and UDM, Fig. 1d&e) does not have the irregularities discussed above for the MSC-I models, and the results are simpler to interpret (Figures S7 & S8, ρ=0). With no gene flow in the data, the estimated migration rates ($\varpi_{AB},\varpi_{BA}$) are close to 0, and the Bayes factor $B_{10}$ is around 1 for the shallow tree (indicating lack of information), and close to or less than 0.01 for medium or deep trees. In other words, these datasets are informative enough that the Bayesian test rejects strongly the alternative hypothesis of gene flow.

In summary, the results of the Bayesian test when there is no model violation are consistent with standard theory (O’Hagan and Forster 2004). It is also interesting to note the differences between the discrete introgression models (BDI and UDI) and the continuous migration models (BDM and UDM). Under BDI and UDI, the biological scenario of no gene flow is represented not only by φ=0 (segment *a* in Table 1(ii), say), but also by φ=1 (segment *c* for UDI), as well as by any arbitrary value over (0,1) (segment *b* for UDI, say). Datasets simulated in this study are not large enough for the Bayesian test to strongly reject the model of gene flow ($H_{1}$) when the test is conducted under the MSC-I models. In contrast, data simulated at medium or high divergences are informative enough to reject $H_{1}$ when the test is conducted under the MSC-M models. Note that strong rejection of the alternative hypothesis is possible with the Bayesian test but impossible with Frequentist tests such as the LRT.

### Performance of Bayesian test: sister populations with recombination

When data were simulated under the null MSC model (Fig. 1a) with recombination (ρ>0) but no selection, and analyzed under the BDI model (Fig. 1b), the Bayesian test is conducted under misspecified models. The false positive rate for detecting introgression was zero at high species divergence (T=16N), mostly zero at medium divergence (40% at ρ=0.0025 and ∼0 at other recombination rates, all at T=4N), but reached 100% at high recombination rates (ρ=0.0025 or higher) and shallow divergence (T=N) (Fig. 2a). Using different cutoff values (ϵ) in the Savage–Dickey density ratio for estimating Bayes factors yielded similar results (Figure S1a). Constraining the population size parameters such that $\theta_{A}=\theta_{X}$ and $\theta_{B}=\theta_{Y}$ or using the same size for all populations yielded similar results although the false positive rate was slightly lower in some cases (Figures S2a, S3a).

These results under the BDI model, in the absence of any selection, mimic those obtained by Smith and Hahn (2024, Fig. 5). Furthermore, our reanalysis of datasets generated by Smith and Hahn (2024) (with ρ=0.025) yielded similar results of high false positives at shallow divergence regardless of selection scheme used (Figure S17a). We suggest that the high false positives reported by the authors are mostly caused by high recombination rates combined with very shallow divergence, rather than by selection.

We then analyzed the data using the simpler unidirectional model with *A*-to-*B* introgression (UDI, Fig. 1c). The results were similar to those obtained under the BDI model (Figures S4–S6). At deep or medium divergences (T=16N or 4N), there were no false positives. At shallow divergence (T=N), the false positive rate was low at ρ=0.0025, about 20% or less, but reached 100% at the two highest values of *ρ* (Figure S4a). Again, constraints on the population size parameters led to similar results but with slightly lower false positive rates, with no false positives at medium and deep divergences (Figures S5a, S6a).

Lastly, we conducted Bayesian test of gene flow using the continuous migration model (BDM or UDM, Fig. 1d&e) as the alternative hypothesis (Figures S7a & S8a). At medium or deep divergences, no false positives were observed when either BDM or UDM was used in the test. At shallow divergence, we obtained high false positives under the BDM model at high recombination rates (with ρ=0.0025 or higher, Figure S7a). At shallow divergence the test under UDM yielded lower false positives than under BDM at ρ=0.0025 but 100% false positives at higher *ρ* (Figure S8a).

In summary, use of the unidirectional models leads to slightly lower false positives than the bidirectional models (BDI and BDM versus UDI and UDM), the constraints on *θ* did not have large effects, but the species divergence time had large effects, with the combination of very recent divergence and very high recombination rates producing excessive false positives in the test.

### Effect of recombination on Bayesian parameter estimation

When there is no gene flow in the data, we expect estimates of the introgression probabilities ($\varphi_{X}$ and $\varphi_{Y}$) and the introgression time ($\tau_{X}$) under the BDI model to fall into five regions of the parameter space: (i) with both $\varphi_{X}$ and $\varphi_{Y}$ close to 0 (or 1) and $\tau_{R}$ matching the true split time (*τ*) and with $\tau_{X}$ unidentifiable; (ii) with one of $\varphi_{X}$ and $\varphi_{Y}$ close to 0 and the other close to 1, and with $\tau_{X}$ matching the true split time (*τ*) and $\tau_{R}$ being unidentifiable; 3) with both $\tau_{X}$ and $\tau_{R}$ matching the true split time, with $\varphi_{X}$ and $\varphi_{Y}$ unidentifiable (see Table 1(i)). Thus in datasets with no signal of gene flow, we expect $\varphi_{X}$ and $\varphi_{Y}$ to vary over the whole range of (0,1) with wide posterior CIs. Our results from the runs that did not detect gene flow support these predictions (Figure S1b).

When there is a false positive, the signal of intralocus recombination is misinterpreted by the model as signals of introgression, and the introgression probabilities ($\varphi_{X}$ and $\varphi_{Y}$) tend to have intermediate posterior means and narrow HPD CIs. For example, at ρ=0.025 and T=N, the estimates of $\varphi_{X}$ and $\varphi_{Y}$ were about 0.2 with tight CIs, leading to strong support for introgression (Figure S1b).

We find that the effective population sizes and species divergence time are always overestimated in the presence of recombination, regardless of whether the test falsely detects gene flow (Figure S1b), with higher recombination rates leading to larger biases in parameter estimates. For example, at ρ=0.025, the estimates of $\theta_{A}$ and $\theta_{B}$ are nearly twice the true values while at ρ=0.25, the estimates are nearly five times the true values. The bias in the estimate of the species split time ($\tau_{R}$) is not as extreme. The ancestral population size ($\theta_{R}$) is underestimated because it is negatively correlated with the species divergence time. We obtained the same pattern of biases when the model was constrained to have $\theta_{A}=\theta_{X}$ and $\theta_{B}=\theta_{Y}$ (Figure S2b) or to have a single population size for all populations (Figure S3b). We observed similar biases in parameter estimates under the UDI model when the test detected introgression (Figures S4b, S5b, S6b).

Under the migration models (BDM and UDM) estimated migration rates ($\varpi_{AB},\varpi_{BA}$) were close to 0 when the test did not detect gene flow, but were nonzero, sometimes with narrow intervals, when the test detected gene flow at shallow divergence (Figures S7b & S8b). At very high recombination rates, all parameters (*θ*, *τ*) involved large biases even if the test did not detect gene flow.

In summary, intralocus recombination leads to an overestimation of the present-day population size parameters, and to a lesser extent, an overestimation of the species divergence time. However, only at shallow divergence do high recombination rates result in consistent nonzero estimates of the introgression probability in the MSC-I models or the migration rate in the MSC-M models, leading to false signals of gene flow.

### Performance of Bayesian test: selection

We examine the effects of selection on the Bayesian test of gene flow by simulating data with no gene flow and no recombination under four different selection schemes: background selection, selective sweep in *A*, selective sweep in *R* and balancing selection. Again the test is conducted under all four models of gene flow (BDI, UDI, BDM and UDM; Fig. 1b–e), with different variants constraining the population sizes (*θ*) under the BDI and UDI models.

We find the false positive rate for the test of gene flow under the BDI model to be low across selection schemes (Fig. 2b; see also Figures S9–S16). An exception is balancing selection at deep divergence, which had a false positive rate of 40%. This pattern was robust to different cutoff values (ϵ) used in the Bayes factor calculation (Figure S9a). We note that in Smith and Hahn (2024), where the data were simulated with recombination, balancing selection also led to high false positive rates of 30–40% (their Figs. 5, S30–S32) but at the medium divergence (T=4N). We confirm this finding in our reanalysis of the data of Smith and Hahn (2024) (Figure S17a).

Unlike the neutral case, constraining the population size parameters such that $\theta_{A}=\theta_{X}$ and $\theta_{B}=\theta_{Y}$ led to 100% false positives in the case of background selection and balancing selection at deep divergence (T=16N; Figure S10a). Similarly, for these two selection schemes, using the same population size parameter for all populations led to 100% false positives at deep divergence and 80–100% false positives at medium divergence (Figure S11a).

When we analyzed the data under the UDI model (Fig. 1c), the false positive rates are generally lower, with no false positives at shallow and medium divergence (Figures S12a, S13a, S14a). At deep divergence, balancing selection has about 30% false positive rate for the model without constraints on *θ* (Figure S12a) while the false positive rate is 80–100% for the two models with constraints on *θ* (Figures S13a, S14a). Background selection caused a high false positive rate of 100% only at deep divergence when all *θ* are constrained to be equal (Figure S14a).

Using the migration models, in particular, the UDM model (Fig. 1d&e) to analyze the data led to almost no false positives (Figures S15a, S16a).

In summary, our results demonstrate that the Bayesian test of gene flow is robust to selective sweeps and background selection while prolonged balancing selection can lead to false signals of gene flow under the discrete MSC-I models. The test under the continuous MSC-M model is highly robust to selection, with virtually no false positives.

### Effect of selection on parameter estimation

When data are simulated under selection with no gene flow and no recombination but analyzed assuming gene flow with no selection, the model is seriously misspecified. If the test of gene flow is significant, examining the estimates may help us to understand the cause of the false positive error. If the test supports no gene flow, the estimates are expected to be close to those under the MSC model, which are still expected to be biased due to impacts of selection.

Under the BDI model, strong background selection (mean selection coefficient 2Ns=−33.25) led to highly reduced estimates of the present-day effective population sizes ($\theta_{A}$ and $\theta_{B}$) and species divergence time ($\tau_{R}$) compared to the neutral case (Figure S9b). The effect on $\theta_{A}$ and $\theta_{B}$ was comparable across the three divergence levels while $\tau_{R}$ was most severely underestimated at deep divergence. The ancestral population size ($\theta_{R}$) was overestimated since it was negatively correlated with $\tau_{R}$. The two selective sweep schemes show no noticeable impact on parameter estimation (Figure S9b). Lastly, balancing selection led to a slight overestimation of $\theta_{A}$ and $\theta_{B}$, and a slight underestimation of $\tau_{R}$, with the strongest effect at the deep divergence (Figure S9b), corresponding to cases with high false positive rates of 20–40% (Figure S9a).

Constraining the population size parameters (either $\theta_{A}=\theta_{X}$ and $\theta_{B}=\theta_{Y}$ or using the same size for all populations) led to more severe biases in parameter estimates (and high false positives) for background and balancing selection (Figures S10b, S11b). Similar patterns were obtained when the data were analyzed under the UDI model (Figures S12b, S13b, S14b) and under the migration models (BDM and UDM, Figures S15b, S16b).

In summary, Bayesian parameter estimation is robust to selective sweeps. Strong background selection leads to underestimation of $\tau_{R}$, $\theta_{A}$ and $\theta_{B}$ while balancing selection leads to overestimation of $\theta_{A}$ and $\theta_{B}$ but reduced $\tau_{R}$, agreeing with known effects of these selective forces (reviewed in Cutter and Payseur 2013). In particular, even if the Bayesian test under the BDM and UDM models is robust to selection and correctly reject gene flow, background selection and balancing selection may cause biased estimates of the species divergence time and population sizes.

### Performance of Bayesian test: number of sequences and sequence length

**Fig. 3.**
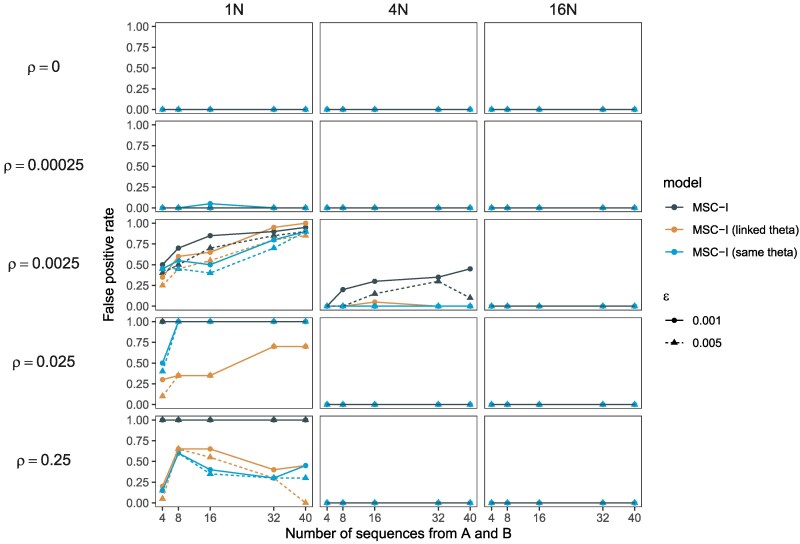
Impact of the total number of sampled sequences (with equal number from each species) on the false positive rate of Bayesian test of gene flow (Ji et al. 2023) under the BDI model (Fig. 1b). Data were simulated under neutral evolution using Ms and Seq-gen. The “linked theta” model enforces the constraints $\theta_{X}=\theta_{A}$ and $\theta_{Y}=\theta_{B}$ (Fig. 1b) while “same theta” refers to the assumption that all populations share the same *θ*.

The results presented above (Fig. 2a) are based on data of 40 sequences (20 from each species). We explored the impact of the number of sequences in the data on the false positive rate for the BDI model (Fig. 1b). We find that using fewer sequences leads to reduced false positives at ρ=0.0025, but at high rates of *ρ* (0.025 and 0.25) the number of sequences has little impact (Fig. 3).

**Fig. 4.**
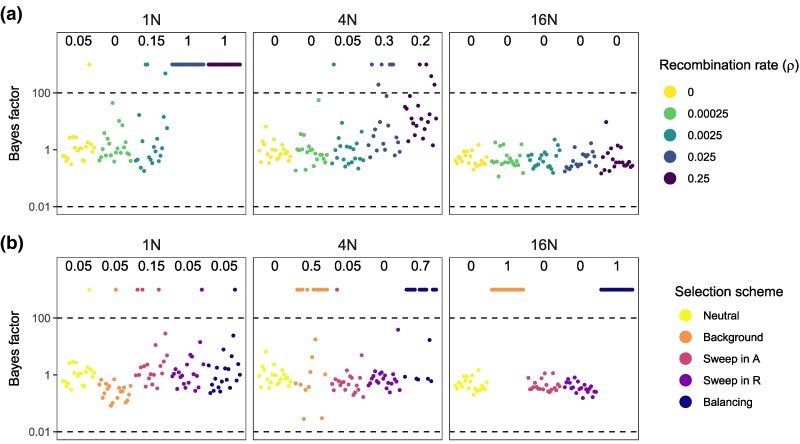
Bayes factors for testing gene flow when the sequence length is 100 bps. Other settings are the same as in Fig. 2 where n=500. See legend to Fig. 2.

We also examined the impact of sequence length. Having shorter sequences with n=100 sites helped to reduce the false positive rate on the shallow tree with recombination (Fig. 4). However for medium and deep divergence, the test appears to become more sensitive to selection and produced even higher false positive rates than at n=500 under some selection schemes.

### Performance of Bayesian test: nonsister populations with recombination

**Fig. 5.**
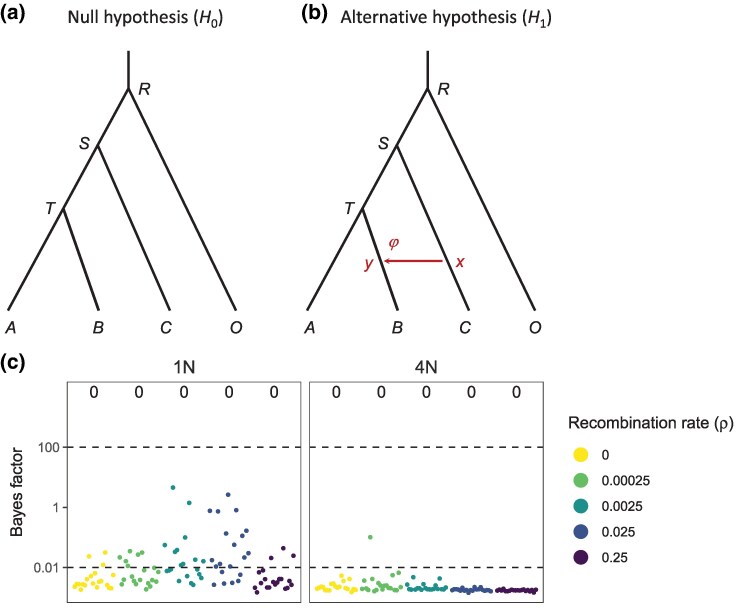
a) Phylogenies for three species (and an outgroup) with no gene flow used to simulate data at different recombination rates with no selection. Parameter values used in the simulation are $\tau_{T}=\frac{1}{4}\theta$, $\tau_{S}=\frac{1}{2}\theta$, $\tau_{R}=5\theta$ for shallow divergence (T=N); and $\tau_{T}=\theta$, $\tau_{S}=2\theta$, $\tau_{R}=5\theta$ for medium divergence (T=4N), with θ=0.005. b) An introgression model with C→B (or x→y) introgression used to analyze the data to assess the false positive of the Bayesian test in presence of recombination. c) Bayes factors for testing the C→B introgression in data simulated under the model of panel a.

The results discussed above concern gene flow between sister populations (Fig. 1). We hypothesize that the false positives for high recombination and shallow divergence reflect the inherent difficulty of detecting gene flow between recently divergent sister populations. We expect the Bayes factor to be more robust for tests of gene flow between nonsister populations. We tested this hypothesis by simulating data under the MSC model of no gene flow on the phylogeny of Fig. 5a, with different recombination rates and no selection. We considered two levels of divergence between *A* and *B*: shallow ($\tau_{T}=\theta /4$ and $\tau_{S}=\theta /2$) and medium ($\tau_{T}=\theta$ and $\tau_{S}=2\theta$), with $\tau_{R}=5\theta$ in both cases, and with θ=0.005. The data were analyzed under the UDI model with introgression of *C* into *B* (inflow; Fig. 5b).

No false positives were observed at any divergence level or recombination rate (Fig. 5c). While there is variation among datasets at the shallow divergence ($\tau_{T}=\frac{1}{4}\theta$), the test is able to strongly reject gene flow, with $B_{10}<0.01$, in most datasets, especially at the high divergence ($\tau_{T}=\theta$).

The robustness of the Bayesian test of gene flow to excessive recombination (in particular when involving nonsisters) is not well-understood. It appears to be widely accepted that if the per-base recombination rate (*r*) and per-base mutation rate (*μ*) are similar, recombination should confound genealogy-based inferences that ignore intra-locus recombination (e.g. Nielsen et al. 2025). Several recombination rates used in our simulations are much higher than the mutation rate (ρ=0.025 and 0.25 correspond to r/μ=ρ/θ=5 and 50). The perceived large impact of recombination on inference in genealogy-based models is thus contradicted by our simulation results and is a misconception.

In the case of one single population, Hein et al. (2005, pp. 148–150) discussed the impacts of recombination on gene genealogies and derived the probabilities that a recombination event may (i) have no effect, (ii) change the branch lengths (coalescent times) but not the gene tree topology, or (iii) change the tree topology. The analysis suggests that a large proportion of recombination events do not change the gene tree topology (e.g. 70% with a sample of n=10; Hein et al. 2005, Table 5.1). The case appears to be similar under the MSC model, as examination of the simulated gene trees suggests that most recombination events cause little or no differences between the gene trees of neighboring segments at the same locus (Zhu and Yang 2021). One may also expect changes of “within-species portions” of the gene tree to have little impact on inference under the MSC. Consider the case of two species (*A* and *B*). If sequences from each species are reciprocally monophyletic on the gene tree, there is no need to invoke migration of sequences for the likelihood model to explain data at the locus, and minor changes to the subtrees within species may not change that interpretation and are unlikely to impact a test of gene flow. This interpretation appears to agree with our finding that the test of gene flow between sisters is far more robust to recombination at deep divergence than at shallow divergence. It may also explain why the test of gene flow between nonsisters is least affected by recombination. We note that our discussions here are speculative, and leave it to future research to investigate the impact of recombination on the distribution of gene-tree topologies and branch lengths under the MSC model and its impact on different inference problems (including detection of gene flow, inference of species trees and estimation of species split times, etc.).

### Potential causes of false positives and biased parameter estimates

Here, we study features of the data and the fitted gene trees to understand how excessive recombination combined with shallow divergence can cause biased parameter estimation and false positives in the Bayesian test of gene flow between sister populations. We note that at the recombination rate of ρ=0.025, there are about 70–80 recombination events on a gene tree for a 500-bp locus at shallow divergence, although recombination may not always change the gene tree topology or branch lengths (Hein et al. 2005, pp. 148–150; Zhu et al. 2022). Recombination can generate chimeric sequences at each locus by combining sequence fragments from multiple ancestors, resulting in an increased time for all sites to reach the common ancestor (Griffiths and Marjoram 1996, 1997). As the MSC model (either with or without gene flow) ignores recombination and assumes a single gene tree for all sites at the locus, one expects recombination to cause elevated coalescent waiting times or branch lengths in the gene tree.

**Fig. 6.**
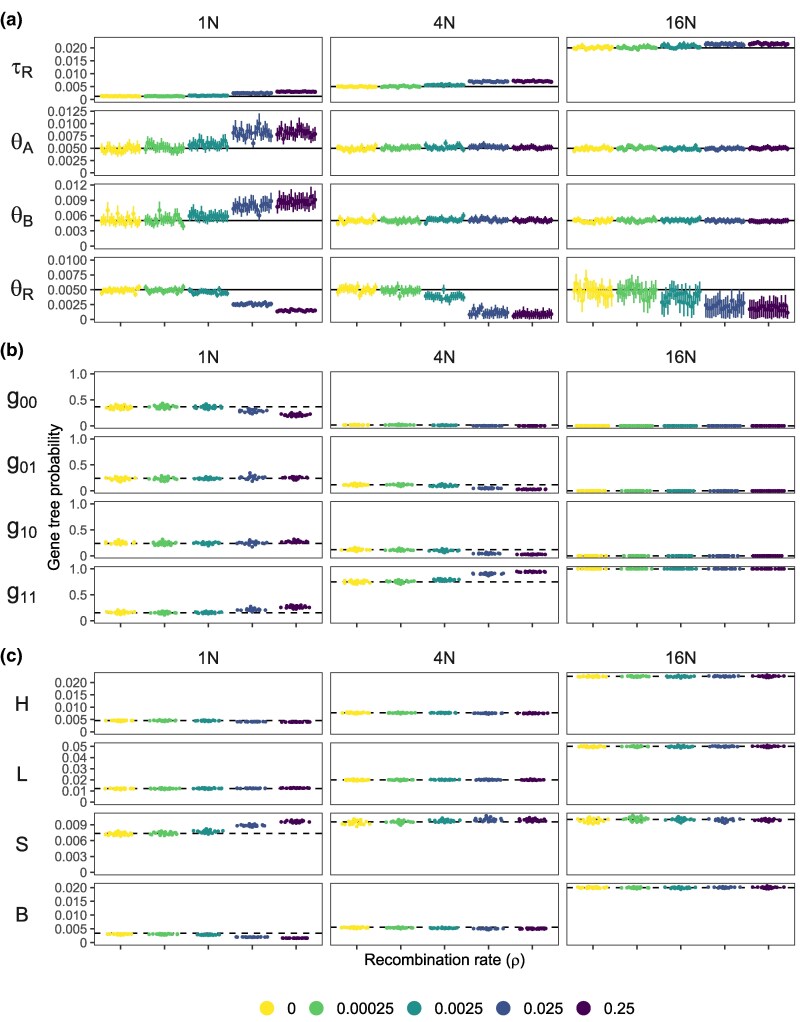
a) Parameter estimates (posterior means and 95% HPD CIs) under the MSC model without introgression (Fig. 1a) using four sequences (two from each species) from 20 simulation replicates. Horizontal lines indicate the true parameter values. b) Probabilities of four types of gene trees classified according to whether the coalescent event of the two sequences from the same species occurs after species divergence. For example, $g_{00}$ means both coalescent events occur in the ancestral population and $g_{01}$ means the two sequences from *A* do not coalesce in *A* while the two sequences from *B* coalesce in *B*. Horizontal dashed lines indicated the expected values when there is no recombination (ρ=0). Analytical expressions of these expected values are in Appendix A. c) Four gene tree statistics, averaged across posterior samples and loci: *H* (height; time to the most recent common ancestor), *L* (length; sum of total branch lengths), *S* (sum of terminal branch lengths) and *B* (average length of the two branches below the root). Horizontal dashed lines indicate the expected values when there is no recombination.

We consider gene trees for a locus with four sequences (two from each species) inferred under the MSC model with no gene flow. First we confirm that parameter estimates at shallow divergence agree with our results from the MSC-I or MSC-M models (Figures S1–S3, S5–S8): $\theta_{A}$, $\theta_{B}$ and $\tau_{R}$ become increasingly overestimated as the recombination rate increases (Fig. 6a). At deep divergence, we see the same effect on $\tau_{R}$ while $\theta_{A}$ and $\theta_{B}$ appear to be unaffected by recombination, because using fewer sampled sequences per species leads to less biased parameter estimates (Figure S18). With 20 sequences per species, $\theta_{A}$ and $\theta_{B}$ were overestimated at all three divergence levels (Figures S1–S3, S4–S8). We calculate the frequencies of four types of gene trees for a locus with four sequences (two from each species), $g_{00},g_{01},g_{10}$ and $g_{11}$, depending on whether the two sampled sequences from each species coalesce within that species. For example $g_{01}$ means the two sequences from *A* do not coalesce in *A* and instead coalesce in the common ancestor (*R*) while the two *B* sequences coalesce in *B* (Fig. 1a). The gene tree here may be considered the best fit under the MSC model which assumes one tree for all sites at the locus. We also calculate four statistics of gene-tree branch lengths from the posterior samples under the MSC model, averaged across loci: the tree height (*H*), the tree length (*L*), the sum of terminal branch lengths (*S*) and the sum of the branch lengths around the root (*B*) (Fig. 6). Analytical expressions for the expected value of these statistics under the simple model of no recombination and no gene flow are in Appendix A.

As the recombination rate increases, the terminal branches become longer and the two branches around the root become shorter without much impact on the tree height and tree length (Fig. 6c), making the gene trees more star-like, apparently because the model is using a single gene tree to explain a mixtures of gene trees. These patterns are consistent with previous findings in a single-population setting (Schierup and Hein 2000). We suggest that those effects on tree shapes with elongated external branches may explain the positive biases in the estimates of $\theta_{A}$, $\theta_{B}$ and $\tau_{R}$ in the Bpp analysis, with larger biases at higher recombination rates (e.g. Figure S1b). Such biases occur at all three divergence levels and under all models of gene flow (Figures S1–S3, S4–S8).

**Fig. 7.**
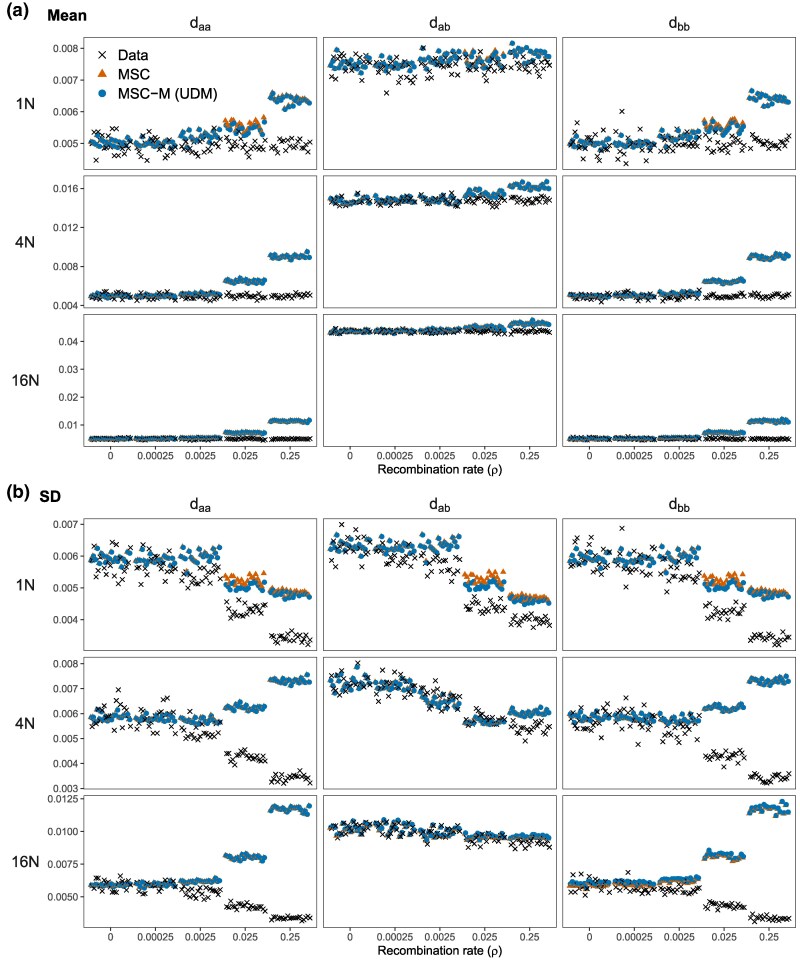
a) Means and b) standard deviations (SDs) in the observed and predicted pairwise sequence distances at different recombination rates for neutral evolution. Observed distances ($d_{aa}$ and $d_{bb}$ within species and $d_{ab}$ between species) are calculated by using a pair of sequences per locus. Predicted distances, under the MSC model with no gene flow and the MSC-M model with unidirectional migration (UDM; Fig. 1e), are calculated using the posterior means of parameters obtained from the Bpp analyses of the data (Figure S8b); see Appendix B.

Note that when recombination is present and ignored, both the null hypothesis of no gene flow ($H_{0}$) and the alternative hypothesis of gene flow ($H_{1}$) are misspecified. To understand why $H_{1}$ fits the data better than $H_{0}$ (thus generating high false positives) at high recombination and shallow divergence, we calculated the means and variances of three pairwise distances ($d_{aa}$ and $d_{bb}$ within species and $d_{ab}$ between species) across loci predicted under $H_{0}$ and $H_{1}$, in comparison with the observed values that are easily calculated using the sequence data. We are able to derive the expected values under the UDM, UDI and BDI models, but not under BDM (see Appendix B). Here, we focus on the UDM model as parameter estimation do not suffer from identifiability issues (unlike BDI and UDI) (Fig. 7). Results for the BDI model are presented in Figure S19, but note that parameters may be poorly estimated in settings where $H_{0}$ fits the data well.

We calculated the observed means and variances of pairwise distances, and used parameter estimates from the Bpp analysis of data generated with intralocus recombination to calculate the expected values under the MSC and UDM models. Regarding the observed means and variances, note that recombination reduces the variance of pairwise sequence distances without altering the mean (Hudson 1983). The combination of high recombination and shallow divergence leads to the largest reduction in the variance $V(d_{ab})$ of $d_{ab}$ across loci. At shallow divergence (T=N), $V(d_{ab})$ at ρ=0.25 is 36.7% that at ρ=0. At deeper divergences (T=4N and 16N), the reduction in $V(d_{ab})$ is 24.5% and 10.2%, respectively. Recombination has a greater effect at more recent divergence. Thus including migration ($\varpi_{AB}>0$) in the UDM model brings the variance of $d_{ab}$ closer to the observed values. Those comparisons suggest that the UDM model of gene flow fits the means and variances of pairwise sequence distances better than the MSC model ($H_{0}$) at recent divergence (T=N) and high recombination rate (ρ=0.025,0.25), cases where high false positives were observed (Figure S8b).

### Bayesian test of gene flow solves the small-data low divergence problem

In the case where there is no gene flow, no recombination and no selection, the false positive rate of the Bayesian test is ∼0 at all three species divergence levels when the test is conducted under any of the four models of gene flow (BDI, UDI, BDM, UDM; Fig. 1b–e) including the three variants on constraining *θ* under the BDI and UDI models (Figure S1–S7, ρ=0 or Figure S15, Neutral; Figure S8, ρ=0 or Figure S16, Neutral). At medium and deep divergences, use of the BDM and UDM models to conduct the test leads to strong rejection of the alternative hypothesis of gene flow. Shallow species divergence alone does not cause false positives for the Bayesian test of gene flow.

Note that when data are simulated under $H_{0}$, the results for the Bayesian test are known for two extremes of the number of loci (*L*): $B_{10}=1$ when L=0, and $B_{10}\to 0$ when L→∞. In small or uninformative datasets, the posterior of parameters (e.g. $\varpi_{AB},\varpi_{BA}$) should be similar to the prior, and $B_{10}$ should be ∼ 1. When L→∞, posterior estimates of $\varpi_{AB},\varpi_{BA}$ converge to the true values (0), and $B_{10}\to 0$ (as $H_{0}$ has fewer parameters than $H_{1}$), leading to strong rejection of gene flow. There exists no theory to expect $B_{10}$ to decrease monotonically from 1 toward 0 when *L* increases from 0 to ∞. It is sufficient if large $B_{10}$ occurs rarely (i.e. if $B_{10}>100$ in no more than <1% of datasets). Judged by this criterion, the Bayesian test is often found to have good Frequentist properties in various testing situations, with the false positive rate below the nominal significance value. This is the case in our simulation examining the Bayesian test of gene flow. Also the Bayesian test is capable of strongly rejecting the alternative hypothesis of gene flow.

It is thus surprising that Cruickshank and Hahn (2014, Fig. 4) found excessive false positives at very shallow divergence, with error rates of up to ∼80% in small datasets of a few loci, when they used the Ima2 program (Hey 2010) to test for gene flow (see also Hey et al. 2015). Note that both Ima and Bpp implement the same MSC-M model (i.e. the isolation-with-migration or IM model), and those simulations are conducted under the MSC model of no gene flow, with no violation of assumptions (cf: Cruickshank and Hahn 2014, p. 3,147). Sethuraman et al. (2025) described this SDLD problem (Hey et al. 2015) as an issue of identifiability, but all parameters in the model are identifiable. Hey et al. (2015) suggested that the false positives may be caused by the use of the integrated likelihood in the LRT implemented in the pioneering work of Nielsen and Wakeley (2001) and Hey and Nielsen (2007).

Our analysis suggests that the high false positives with the LRT implemented in Ima reported by Cruickshank and Hahn (2014) and Hey et al. (2015) are due to inappropriate use of the integrated likelihood, the poor choice of priors, and the general difficulty of using a Bayesian high-dimensional MCMC program to conduct maximum likelihood (ML) analysis. To see the difference between the (traditional) LRT and the LRT* based on integrated likelihood, let the parameter vector for the alternative hypothesis $H_{1}$ be θ=(ω,λ), where *ω* are the parameters of interest and *λ* are the nuisance parameters. In the case of the MSC-M model for two species, $\omega =(\varpi_{AB},\varpi_{BA})$ and $\lambda =(\tau_{R},\theta_{A},\theta_{B},\theta_{R})$ (Fig. 1d). The null hypothesis $H_{0}$ is represented by the parameters of interest taking special values: $\omega =\omega_{0}=(0,0)$. Given the parameters θ, sequence data at multiple loci are i.i.d., and constitute data points in the MSC model.

In so-called “stable-estimation” problems, where each data point depends on the whole parameter vector θ, and the number of data points far exceeds the number of parameters, the standard practice is to optimize nuisance parameters in calculation of the likelihood values under both the null and alternative hypotheses (Stuart et al. 1999, Chapters 21 & 22, in particular § 22.7). In other words, the likelihood ratio test statistic is defined as


(1) $$2\Delta \ell =2\log\;\frac{L_{1}(\hat{\omega },\hat{\lambda })}{L_{0}(\hat{\lambda })},$$

where the likelihood values under both models, with $L_{0}(\lambda )=L_{1}(\omega_{0},\lambda )$, are calculated at their maximum likelihood estimates (MLEs). This is the approach taken by Zhu and Yang (2012) and Dalquen et al. (2017) in their LRT for migration under the MSC-M model (see also Lohse et al. 2011). The approach integrates over the gene tree topologies and branch lengths at the loci numerically to calculate the likelihood function, but parameters (ω,λ) are optimized via a numerical optimization algorithm. The program (3s), however, has the limitation of being able to handle at most three sequences at each locus. Note that gene trees at multiple loci are random variables and always integrated out.

The Ima3 program (Hey 2010; Hey et al. 2015, 2018) implements the MSC-M or IM model in the Bayesian framework, and generates samples $\left(\omega^{\left(k\right)},\lambda^{\left(k\right)}\right)$ from the joint posterior p(θ|x). The authors chose to summarize $\left(\omega^{\left(k\right)}\right)$ while ignoring $\lambda^{\left(k\right)}$, thus estimating the marginal distribution p(ω|x), effectively integrating out the nuisance parameters (*λ*). Note that even if no explicit step of integration is taken, the sampled values of *ω* (with the sampled values of *λ* ignored) are a sample from the marginal distribution, p(ω∣x). Then with uniform priors,


p(ω,λ)=p(ω)p(λ|ω)∝1,

the estimated marginal posterior density is treated as the integrated likelihood


(2) $$L_{1}^{*}(\omega )=\frac{\;p(\omega \;|\;x)}{p(\omega )}=\int p(x\;|\;\omega ,\lambda )p(\lambda \;|\;\omega )\;d\lambda ,$$

where $p(x\;|\;\omega ,\lambda )\equiv L_{1}(\omega ,\lambda )$ is the traditional likelihood. Calculation of the likelihood value under the alternative hypothesis, $L_{1}^{*}(\hat{\omega })$, thus involves estimating the density p(ω|x) and finding its optimizer $\hat{\omega }$. Similarly the likelihood under the null hypothesis is an integral over the nuisance parameters (*λ*),


$$L_{0}^{*}=\int p(x\;|\;\omega =0,\lambda )p(\lambda \;|\;\omega =0)\;d\lambda .$$

The (integrated) likelihood ratio test (LRT*) is then based on the statistic


$$2\Delta \ell^{*}=2\log\;\frac{L_{1}^{*}(\hat{\omega })}{L_{0}^{*}}.$$

While some statisticians argue for the advantage of the integrated likelihood as an approach to dealing with nuisance parameters (Berger et al. 1999), the idea appears to be proposed mostly to deal with complex or irregular situations. For example, in all examples of Berger et al. (1999) for illustrating the usefulness of the integrated likelihood, there exist either as many nuisance parameters as data points, or only one sampling replicate from the probability distribution indexed by the nuisance parameter.

Besides the use of integrated likelihood, a few other implementation features of the Ima program may have contributed to the problem of excessive false positives in the LRT*. First, uniform priors on parameters (θ,τ, and ϖ) used in Ima are highly informative but implausible. With the uniform prior, a large upper bound is often used to avoid excluding unlikely but not impossible values, so that the prior mean may be too large. The integrated likelihood is well-known to be sensitive to the upper bound in the uniform prior (see Fig. 6.6 in Yang 2014 for an example). In the case of improper priors, marginal likelihood values may even be infinite (Dawid et al. 1973). Thus uniform priors, once described as “uninformative,” are here misinformative. Second, the predefined binning and estimation of multidimensional densities using empirical histograms may not be reliable. The bins for parameters are determined before the MCMC by using the priors, and the program collects sampled parameter values into those bins. If the prior and the posterior are very different, the binning strategy may be inadequate so that all sampled values may be lumped into one bin, and the run is wasted. A simple fix may be to save the sampled values and summarize the sample after the MCMC. Third, estimating the multi-dimensional density and finding its maximum using the MCMC sample appears to be too challenging a task to be practical. Fourth, Ima defines parameters *θ* and *τ* using a per-locus mutation rate. This parametrization means that the priors assigned on *θ* and *τ* assumes higher per-base mutation rates for shorter loci, which does not appear plausible in analysis of genomic sequence data. This is an issue whether or not mutation rates are allowed to vary among loci. In programs such as G-phocs (Gronau et al. 2011) and Bpp, the per-base mutation rate is used, so that the prior assumes comparable per-base mutation rates among loci. Finally, there may be issues with the MCMC algorithm implemented in Ima3, an improved version of Ima2 (Flouri et al. 2023, Fig. 4).

**Fig. 8.**
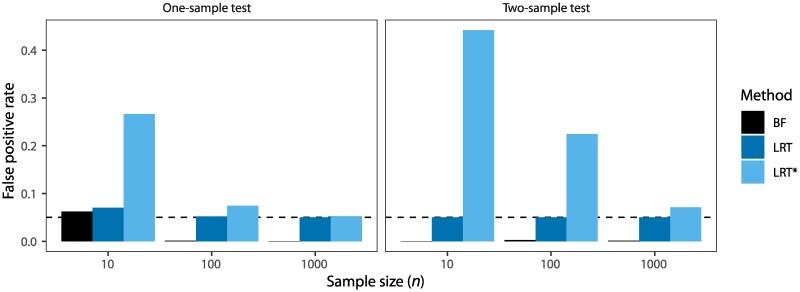
False positive rate at the 5% level for three tests (Bayes factor, LRT, and LRT*) applied to a) the one-sample test and b) the two-sample test of Appendix C, calculated by simulating $10^{6}$ datasets under the null hypothesis. A false-positive error occurs if the LRT statistic (equations (C5), (C7), (C13) & (C17)) is >3.84, or if $B_{10}>19$ in the Bayesian test (equations (C4) & (C12)). For the one-sample test, we used k=10, and α=10,β=0.5 in the prior, while for the two-sample test, we used μ=1 for the data, and τ=10 in the prior.

In Appendix C, we use the one-sample test (which compares $H_{0}\;:\;\mu =0$ against $H_{1}\;:\;\mu \neq 0$ under the normal model with the unknown variance $\sigma^{2}$ being the nuisance parameter) and the two-sample test ($H_{0}\;:\;\mu_{1}=\mu_{2}$ and $H_{1}\;:\;\mu_{1}\neq \mu_{2}$ with known variance) to illustrate the differences among the Bayesian test, the (traditional) LRT, and the integrated likelihood ratio test (LRT*) (Fig. 8). Note that conducted at the 5% significance level, the type-I error rate for the LRT is exactly 5%, but differs considerably from 5% for the LRT* and the Bayesian test. When the data size n→∞, the false positive rate approaches 5% for the LRT and LRT*, and 0 for the Bayesian test. In small datasets with poorly chosen priors, the LRT* can generate excessive false positives, while the Bayesian test in general has false positive rate below 5%.

In summary the high false positives at shallow divergence for the test using Ima discussed by Cruickshank and Hahn (2014) and Hey et al. (2015) do not apply to the Bayesian test (Flouri et al. 2023; Ji et al. 2023) or the (traditional) LRT (Zhu and Yang 2012; Dalquen et al. 2017). They are a consequence of the use of the integrated likelihood and misinformative uniform priors in the Ima program (Nielsen and Wakeley 2001; Hey and Nielsen 2007). The so-called small data-low divergence (SDLD) problem (Hey et al. 2015) can be solved by using a Bayesian test (Ji et al. 2023) and more plausible priors.

### Impact of parameter values used in simulation and the procedure of Frequentist simulation

The performance of a hypothesis test such as the Bayesian test of gene flow (Ji et al. 2023) may depend on the values of parameters. For example, the species split time appears to have a large impact on the false positive rate and power of the test of gene flow between sister populations with recombination (Yang et al. 2025 and this study). Here, we discuss the biological realism of parameter values used in this study (see Smith and Hahn 2024).

The population size parameter may vary among species by orders of magnitude, with θ=0.001 to be a relatively small value (typical of humans, Patterson et al. 2012) and 0.01 to be a large value (typical of insects such as mosquitoes and butterflies, Thawornwattana et al. 2018, 2023a). The value used here (θ=0.005) lies between those extremes. Similarly the divergence time for a pair of species may vary hugely among biological systems. Note that the average coalescent waiting time for two sequences sampled from the same population of size *N* is 2N. The shallow divergence used (T=N), with the species split time to be half the average coalescent time, represents very recent divergence. The population recombination rate ρ=4Nr depends on the effective population size *N* and the recombination rate per base per generation, both varying considerably among species. The low rate (ρ=0.00025) is typical for humans. The high rate (ρ=0.25) may be realistic for some social insects with very large population sizes and high recombination rates (DeLory et al. 2024) but is too large for mammals (see Zhu et al. 2022; Yan et al. 2023 and references therein). The selection coefficient is expected to vary hugely among species and across the genome. Background selection simulated in this study, at 2Ns=−33.25 (Smith and Hahn 2024), appears to be very strong as a new mutant with Ns≪−1 is effectively “dead on arrival.” This was chosen based on estimated selection coefficients for nonsynonymous substitutions in *Drosophila* (Huber et al. 2017) and may not be representative of organisms with much smaller population sizes or of background selection due to loose linkage to other functional elements in the genome (Charlesworth and Jensen 2021).

It should be pointed out that Smith and Hahn (2024) did not conduct the Frequentist simulation correctly. In their simulation under models of introgression (which we did not address as all our simulations were conducted under the null model of no gene flow), the authors generated loci and replicate datasets using random values of introgression time and introgression probability ($\tau_{X}$ and $\varphi_{Y}$ of Fig. 1b). Here, both $\tau_{X}$ and $\varphi_{Y}$ are parameters in the model and should be fixed when replicate datasets are generated. Frequentist properties of a hypothesis test (such as the false positive rate and power) or of an estimation method (such as the bias and variance) are defined with the values of parameters in the model fixed. For example, the false positive rate is the probability of rejecting the null hypothesis in replicate datasets, all of which are generated under the null model with the same set of parameter values, even though a respectful test should have the false positive rate under control whatever the parameter values are. Second, when simulating selective sweeps and background selection, Smith and Hahn (2024) sampled the middle 500 base pairs in each 10kb region, with the site under selection to be in the center of the region. This way the selected site is always included in the 500-bp sampled segment. When multilocus sequence data are compiled for analysis under the MSC model, we expect short segments to be sampled at random from the genome, and may not include the selected site. For example, a noncoding segment sampled at random from the genome may not necessarily be close to an exon. Unfortunately both sampling parameter values from a distribution for each data replicate and fixing random variables at particular values for all replicates are very common mistakes in simulations in molecular phylogenomics. Finally, Smith and Hahn (2024) conducted the test by examining whether the 95% HPD CIs for introgression probabilities include the null value 0. This approach may be very sensitive to the prior on parameters. If the dataset is uninformative, the posterior 95% CI will be very close to the prior CI, which excludes 0, so that the test will have a false positive rate of ∼100% when the data are uninformative. The standard approach is to use the Bayes factor (Ji et al. 2023 ).

Nevertheless, we note that incorrect simulation and incorrect application of the Bayesian test are not the main causes for the high false positives for the Bayesian test of gene flow between sister species reported by Smith and Hahn (2024). In our simulations we find similar high false positives for the test of gene flow between sister populations in the setting of shallow divergence and high recombination.

## Conclusions

In this study, we have examined a number of factors that may influence the Bayesian test of gene flow, including the size of the dataset, the assumptions about the population sizes on the phylogeny, the assumed mode of gene flow (MSC-I versus MSC-M), the direction of gene flow, as well as intralocus recombination and natural selection. Below we provide a summary of our major findings.

- Recombination causes false positives in the Bayesian test of gene flow between sister species only when species divergence is shallow (with divergence time shorter than the expected coalescent time) and the population recombination rate (*ρ*) is very high. Otherwise, the test is robust (Fig. 2a, Figures S1a–S8a). Ignoring recombination inflates contemporary population-size and divergence-time estimates while reducing ancestral population sizes estimates and can mimic weak introgression (Figures S1b–S8b).
- Bayesian test of gene flow between nonsister species has very low false positives even in presence of excessive recombination (Fig. 5c).
- Sensitivity to high recombination for the test of gene flow between sisters at shallow divergence may be ameliorated by using a smaller number of sequences for each species (Fig. 3 & Figure S18) or by having shorter sequences at each locus (Fig. 4). One should however note that larger datasets with more samples per species and with longer sequences improve the precision of parameter estimation. It may be useful to examine whether parts of the genome under different influence from recombination, such as coding and noncoding parts of the genome, provide consistent signals of gene flow (Thawornwattana et al. 2022, 2023b). If possible, including an additional population that transforms a sister into a nonsister population to make gene flow become between nonsister populations will be effective at reducing false positives in gene flow tests.
- Natural selection at reasonable strengths have minimal effects on the test of gene flow between sister populations, except for prolonged balancing or background selection at deep divergence under the discrete introgression models (Fig. 2b, Figures S9–S16), possibly because persistent ancestral polymorphism might be misinterpreted by the Bayesian test as signals of gene flow. These results contradict the claim of Smith and Hahn (2024), that selection causes false positives in tests for gene flow between sister species. Instead, the high false positive rates they reported are primarily caused by high recombination rates combined with very shallow divergence.
- High recombination makes gene trees appear more star-like (Fig. 6) and reduces variance among loci (Fig. 7 & Figure S19), biasing parameter estimates and producing false signals of gene flow, especially at shallow divergence.
- Without recombination or selection, shallow divergence alone does not lead to false positives for the Bayesian test of gene flow between sister species (Figs. 2–4, Figure S1–S8, ρ=0). Excessive false positives of the LRT using Ima3 in such scenarios reported by Cruickshank and Hahn (2014) and Hey et al. (2015) are due to use of the integrated likelihood and misinformative uniform priors in the likelihood ratio test (Fig. 8 and Appendix C).
- The other factors examined in our simulation, such as different constraints among population-size parameters on the phylogeny, the assumed mode of gene flow (the discrete MSC-I versus continuous MSC-M models), the direction of gene flow (unidirectional versus bidirectional models) have less important impacts on the false positives of the Bayesian test of gene flow. The different model specifications may be useful when one examines the robustness of the test in analysis of empirical data.

Overall, we find that in most scenarios examined the Bayesian test has very low false-positive rates. The test is particularly robust to selection and recombination when conducted under the unidirectional continuous migration model (UDM), or if gene flow being tested involves nonsister lineages on the phylogeny.

This study has focused on false positive rates of the Bayesian test under the null model of no gene flow, but a number of recent studies have also examined the power of the test to detect introgression/migration, by simulating data under alternative models that include gene flow of various forms. Huang et al. (2020) examined the information content in multilocus datasets for inference under the MSC model either with or without gene flow, and found that among many factors that affect the information content in the data (such as the number of loci, the number of sequences sampled per species, the sequence length, and the mutation rate), the number of loci is often the most important, although to infer gene flow the use of multiple sequences per species, in particular, from the hybridizing species, is important. Thawornwattana et al. (2023a) examined the inference of gene flow with the direction misspecified (e.g. with X→Y introgression assumed when introgression is in fact from Y→X) and found that the use of the bidirectional model can lead to reliable inference of the direction of gene flow. Huang et al. (2022) and Thawornwattana et al. (2025) examined the inference of gene flow when the mode of gene flow is misspecified (e.g. when the assumed model is the discrete MSC-I model when in fact gene flow is continuous as specified under the MSC-M model), finding that reliable inference of gene flow and estimation of parameters such as species split times is often possible despite the misspecified mode. Ji et al. (2025) assessed the impact of genotyping errors at low read depths and found that inference of gene flow is reliable at read depths of 10x or higher using today’s sequencing technologies. These simulations have improved our understanding of the statistical properties and behaviors of full-likelihood methods for inferring gene flow under a variety of conditions. Notably recent advancements in MCMC algorithms (Flouri et al. 2020, 2023) have made it possible to apply the MSC models with gene flow to datasets of ∼10,000 loci (albeit on a small phylogeny with no more than a dozen species) which are large and informative enough to allow meaningful inference of gene flow (e.g. Flouri et al. 2023; Thawornwattana et al. 2023a). We conclude that full likelihood methods for testing gene flow and estimating its rate as implemented in Bpp can reliably be applied to infer gene flow both between sister populations and between nonsister populations using genomic data.

## Materials and methods

### Simulation of multilocus data under the MSC with recombination or selection

In the first set of simulations, we used different recombination rates but no selection (and no gene flow): ρ=0, 0.00025, 0.0025, 0.025, and 0.25. The highest rate may be plausible for some species of social insects (e.g. DeLory et al. 2024). Note that Smith and Hahn (2024) used a single high recombination rate of ρ=0.025.

We simulated multilocus sequence data under a two-species model with no gene flow (Fig. 1a). The model has three populations (present-day populations A,B and their common ancestor *R*) and two parameters: a species divergence time (*τ*) and a population size parameter (*θ*) assumed to be identical for all three populations. Here, τ=Tμ and θ=4Nμ, where *T* is the divergence time in generations, *μ* is the neutral mutation rate per site per generation and *N* is the effective population size. Both parameters are in units of the expected number of mutations per site. We fixed θ=0.005 and used τ=θ/4, *θ* and 4θ (or T=N, 4N and 16N generations) to represent shallow, medium and deep divergence, respectively. The sequence data consist of 500 independent loci. Each locus contains 40 sequences (20 from each species), with the sequence length to be 500 sites. There were 20 replicates for each parameter setting. In total 300 datasets were simulated (for 5 recombination rates, 3 divergence times, and 20 replicates).

For some settings both forward simulation using Slim v4.0.1 (Haller and Messer 2023) and backward coalescent simulation using Ms (Hudson 2002) and Seq-gen (Rambaut and Grassly 1997) were used and noted to produce nearly identical results. Then Ms was used as it was much faster than forward simulation by Slim.

In the second set of simulations, we used Slim to perform forward simulation under five selection schemes but no recombination (and no gene flow): neutral evolution, background selection, selective sweep in *A*, selective sweep in *R* and balancing selection. We followed the simulation procedure of Smith and Hahn (2024) with two small modifications (see below). The simulated history at the selected site was recorded as a tree sequence for sampled individuals. Neutral mutations at linked sites at the locus were subsequently added using Pyslim v1.0.3 (Haller et al. 2019) and Tskit v0.5.5 (Kelleher et al. 2018). The length of the region in Slim simulation was 10 kilobases (kb) but only the middle 500 positions were used in analysis (which may and may not include the site under selection). We followed Smith and Hahn (2024) to rescale the population size by 100 to reduce the computational load of forward simulation, while rescaling mutation rate (*μ*), recombination rate (*r*), and selection coefficient (*s*) so that population parameters Nμ, *Nr*, and *Ns* remain unchanged. Similarly, species divergence times and migration times were scaled down by a factor of 100. For background selection, 75% of the new mutations are deleterious with a selection coefficient (*s*) drawn from a gamma distribution with mean −0.0133 and shape parameter α=0.35, and a dominance coefficient h=0.25. We used N=1,250 individuals in the simulation (i.e. N=125,000 before the scaling), which gives 2Ns=−33.25. For selective sweeps, a beneficial mutation has a selection coefficient (*s*) drawn uniformly from the interval [0.1,0.5] and a dominance coefficient h=1. The simulation was discarded if the beneficial mutation was lost. For balancing selection (i.e. frequency-dependent selection), a mutation is beneficial when it is rare and deleterious when it is common. Let *p* be the frequency of allele *A*. Then the fitness values of the three genotypes AA,Aa, and *aa* were 1.5−p, $1.25-\frac{1}{2}p$, and 1, respectively. When p=1, the fitness values were 0.5, 0.75, and 1.

We modified the simulation procedure of Smith and Hahn (2024) (with scripts available at https://github.com/meganlsmith/selectionandmigration) as follows. First, in the two selective-sweep schemes, we sampled a site under selection at random along the sequence rather than fixing it to be in the middle of the sequence. Second, under balancing selection (frequency-dependent selection), we assume no dominance ($h=\frac{1}{2}$) whereas Smith and Hahn (2024) assumed complete dominance. For the two selective-sweep schemes and balancing selection, we fixed the proportion of loci under selection to 10% while background selection affected all loci. In total 240 datasets were simulated (for 4 selection schemes excluding neutral evolution, 3 divergence times, and 20 replicates).

As variations to our standard simulation, we simulated data under neutral evolution at the five different recombination rates, but the number of sampled sequences per locus is S=4,8,16,32 and 40 (with an equal number per species). There were 300 datasets in total (5 sample sizes, 3 divergence times, 20 replicates). We also included a set of simulations with the sequence length of 100 sites (cf: 500 sites in the standard simulation).

### MCMC analysis of simulated data using Bpp

The simulated datasets were analyzed under both MSC-I and MSC-M models with either bidirectional or unidirectional gene flow (Fig. 1b–e), to calculate the Bayes factor to conduct the Bayesian test of gene flow (Ji et al. 2023). The bidirectional introgression (BDI, Fig. 1b) model involves nine parameters: five population sizes ($\theta_{A},\theta_{B},\theta_{X},\theta_{Y}$, and $\theta_{R}$), one species split time ($\tau_{R}$), one introgression time ($\tau_{X}$) and two introgression probabilities ($\varphi_{Y}$ for A→B introgression and $\varphi_{X}$ for B→A introgression). We also analyzed the data using two variants of this BDI model, assuming either $\theta_{A}=\theta_{X}$ and $\theta_{B}=\theta_{Y}$ or a single *θ* for all populations. Additionally, we analyzed the data using a unidirectional model (UDI, Fig. 1c), with introgression from *A* to *B* forward in time, using the same sets of constraints on *θ*s. Finally, we analyzed the data under the continuous migration model (MSC-M), assuming either bidirectional or unidirectional migration (BDM and UDM, Fig. 1d–e). The BDM model has six parameters: three population sizes ($\theta_{A},\theta_{B}$, and $\theta_{R}$), one species split time ($\tau_{R}$) and two migration rates ($\varpi_{AB}$ for A→B migration and $\varpi_{BA}$ for B→A migration) while the unidirectional model has five parameters (no $\varpi_{BA}$). Here, $\varpi_{AB}=\frac{m_{AB}}{\mu }=\frac{4M_{AB}}{\theta_{B}}$ is the mutation-scaled migration rate (i.e. when one time unit is the expected amount of time needed to accumulate one mutation per site), where $m_{AB}$ is the proportion of immigrants in the recipient population *B* from *A* per generation (with time running forward) and $M_{AB}=N_{B}m_{AB}$ is the expected number of A→B migrants per generation.

We assigned gamma priors on *θ* and $\tau_{R}$ with prior means matching the true values: θ∼G(2,400) for all populations (mean 0.005), $\tau_{R}∼G(2,1,600)$ when the true divergence time is τ=θ/4 (mean 0.00125), $\tau_{R}∼G(2,400)$ when τ=θ (mean 0.005), and $\tau_{R}∼G(4,200)$ when τ=4θ (mean 0.02). For the introgression model (Fig. 1b&c), both introgression probabilities ($\varphi_{X}$ and $\varphi_{Y}$) were assigned the uniform prior U(0,1). The age of the root and the introgression time ($\tau_{R},\tau_{X}$) were assigned a uniform-Dirichlet prior (Yang and Rannala 2010, eq. 2), with $\beta =\tau_{X}/\tau_{R}∼U(0,1)$. For the migration model (Fig. 1d&e), both migration rates ($\varpi_{AB},\varpi_{BA}$) were assigned the gamma prior G(2,1) with mean 2.

We used Bpp v4.8.2 (Flouri et al. 2020, 2023) to perform posterior inference. For each dataset, we ran two chains of MCMC, each with initial 64,000 iterations of burn-in followed by $10^{6}$ iterations of the main chain, recording samples every 100 iterations. Convergence was assessed by comparing posterior estimates from the two runs. Samples from convergent runs were combined to produce final posterior summaries and to calculate the Bayes factor. Each MCMC run took about 70 hours for the MSC-I model (Fig. 1b&c) and about 120 hours for the MSC-M model (Fig. 1d&e).

Unlike Smith and Hahn (2024), we did not use the locusrate and heredity options in Bpp (which allow for variation in the rate and ploidy among loci), to avoid computational issues from fitting too many parameters in the model when data were from only two species (Fig. 1a). We reanalyzed all datasets generated by Smith and Hahn (2024) (assuming the high recombination rate of ρ=0.025 with different selection schemes) under the BDI model using the prior and MCMC setting as above to confirm that these two options did not impact their results. There were 660 datasets in total (5 selection schemes, 3 divergence times, 20 replicates, with three of the five selection schemes having three proportions of loci under selection).

### Calculation of the Bayes factor for testing gene flow under four different MSC models of gene flow

A false positive occurs when the Bayes factor in support of gene flow $B_{10}>100$ so that the null hypothesis of no gene flow ($H_{0}$) is strongly rejected. The false positive rate is thus estimated by the proportion of simulated replicate datasets in which $B_{10}>100$. We calculated $B_{10}$ for each simulated dataset using the Savage–Dickey density ratio (Dickey 1971; Ji et al. 2023).

We note that the test comparing the null hypothesis of no introgression ($H_{0}$, Fig. 1a) against the alternative hypothesis of BDI ($H_{1}$, Fig. 1b) has a number of nonstandard features; see Yang et al. (2025) for details. Here, we provide an overview. In regular cases, $H_{0}$ should correspond to one point (called the ‘null point’) in the parameter space for $H_{1}$. However, here in the test of MSC with no gene flow (Fig. 1a) against the BDI model (Fig. 1b), the null point is not one point but instead consists of five lines or planes in the parameter space of $H_{1}$ (Table 1(i)). For example, while the line specified by $\varphi_{X}=0$ and $\varphi_{Y}=0$ with introgression time $\tau_{X}$ being indeterminate represents the biological scenario of no gene flow, the line $\varphi_{X}=1,\varphi_{Y}=1$ with $\tau_{X}$ indeterminate also represents no gene flow. We define a “null region” (∅) around the null point in the parameter space of $H_{1}$, within which introgression is negligible, using two small cutoff values $\epsilon_{\varphi }$ (for φ) and $\epsilon_{\beta }$ (for $\beta =\tau_{X}/\tau_{R}$),


(3) $$\begin{array}{rl} ⊘ & =\{\varphi_{X}<\epsilon_{\varphi },\varphi_{Y}<\epsilon_{\varphi }\}\\ & \cup \{1-\varphi_{X}<\epsilon_{\varphi },1-\varphi_{Y}<\epsilon_{\varphi }\}\\ & \cup \{1-\frac{\tau_{X}}{\tau_{R}}<\epsilon_{\beta }\}\\ & \cup \{\varphi_{X}<\epsilon_{\varphi },1-\varphi_{Y}<\epsilon_{\varphi }\}\\ & \cup \{1-\varphi_{X}<\epsilon_{\varphi },\varphi_{Y}<\epsilon_{\varphi }\} \end{array}$$

(Yang et al. 2025). $B_{10}$ is then given by the Savage-Dickey density ratio, approximated by


$$B_{10,\epsilon }=\frac{P(\emptyset )}{P(\emptyset |x)}=\frac{4\epsilon_{\varphi }^{2}+\epsilon_{\beta }}{P(\emptyset |x)},$$

that is, the ratio of the prior and posterior probabilities that the parameters are in the null region. We used $\epsilon_{\varphi }=\epsilon_{\beta }=\epsilon =0.001,0.005$ and 0.01, to confirm that the result is not sensitive to ϵ. As we used the uniform prior U(0,1) on $\varphi_{X},\varphi_{Y}$ and *β*, the prior probability is simply $P(\emptyset )=4\epsilon^{2}+\epsilon$, while the posterior probability P(∅|x) was calculated as the proportion of the posterior MCMC samples in which equation (3) holds.

For the UDI model (Fig. 1c, Table 1(ii)), the null region is defined as


(4) $$\emptyset =\{\varphi_{Y}<\epsilon_{\varphi }\}\cup \{1-\frac{\tau_{X}}{\tau_{R}}<\epsilon_{\beta }\}\cup \{1-\varphi_{Y}<\epsilon_{\varphi }\}.$$

Then $B_{10}\approx P(\emptyset )/P(\emptyset \;|\;x)$, with P(∅)=3ϵ and with P(∅|x) estimated from the posterior MCMC samples.

We also conducted the Bayesian test of gene flow under the continuous migration models (BDM and UDM, Fig. 1d&e) (Ji et al. 2023). In case of BDM, the hypotheses are $H_{0}\;:\;\varpi_{AB}=0$ and $\varpi_{BA}=0$ and $H_{1}\;:\;\varpi_{AB}>0$ and $\varpi_{BA}>0$. We then define the null region as


(5) $$\emptyset =\{\varpi_{AB}<\epsilon ,\varpi_{BA}<\epsilon \}.$$

In the case of UDM model (Fig. 1e), the null region is


(6) $$\emptyset =\{\varpi_{AB}<\epsilon \}.$$

We used ϵ=0.0454,0.1035, and 0.1486, corresponding to the tail probabilities α=G(ϵ;2,1)=0.001, 0.005 and 0.01, where G(ϵ;2,1) is the cumulative distribution function (CDF) of the gamma prior G(2,1). The prior probability P(∅) is then $\alpha^{2}$ for the BDM model and *α* for the UDM model (Fig. 1d&e). For example, for the test under BDM, $B_{10}\approx \frac{P(\emptyset )}{P(\emptyset \;|\;x)}=\frac{\alpha^{2}}{P(\emptyset \;|\;x)}$, with P(∅|x) estimated by the proportion of MCMC samples in which $\varpi_{AB}<\epsilon$ and $\varpi_{BA}<\epsilon$.

We note that Smith and Hahn (2024) calculated the false positive rate for Bpp using the 95% HPD CI, with a false positive if the CIs of $\varphi_{X}$ or $\varphi_{Y}$ did not include 0. It may be possible to generate useful test results using the HPD CI, but care is needed concerning the prior on φ (Ji et al. 2023). The U(0,1) prior has the 95% CI 0.025–0.975 so that the test based on the HPD CI has a false positive rate of 100% when the data are uninformative.

### Simulation to test gene flow between nonsister species

We simulated data under the MSC model using a phylogeny for three species (with an outgroup) (Fig. 5a), using different recombination rates but assuming no gene flow. Each dataset consists of 500 independent loci, with 31 sequences per locus (10 for each ingroup species and 1 for the outgroup), and with the sequence length to be 500 sites. The number of replicates was 20 at each recombination rate. Other settings are the same as in the case of two species (Fig. 2a).

Each dataset was analyzed under the model of Fig. 5b with C→B (or x→y) introgression to test for gene flow. The null hypothesis of no introgression $H_{0}$ (Fig. 5a) is represented by the parameter of interest φ taking the null value $\varphi_{0}=0$ in the alternative hypothesis $H_{1}$ (Fig. 5b). While the null value $\varphi_{0}=0$ is at the boundary of the parameter space for $H_{1}$, and furthermore when $\varphi =\varphi_{0}$, the introgression time $\tau_{X}$ is unidentifiable. Those irregularities do not pose a problem to the use of the Savage-Dickey density ratio to compute the Bayes factor (Ji et al. 2023). We define the null region as ∅={φ<ϵ}. Then $B_{10}\approx \frac{P(\emptyset )}{P(\emptyset \;|\;x)}=\frac{\epsilon }{P(\emptyset \;|\;x)}$, with P(∅|x) estimated from the posterior MCMC sample.

## Supplementary Material

msaf327_Supplementary_Data

## Data Availability

Simulated datasets and scripts for simulation and Bpp analysis are available on Zenodo at https://doi.org/10.5281/zenodo.17019348.

## Appendix A. Gene-tree properties under the MSC model of no gene flow in the case of 2+2 sequences

Consider the MSC model for two species (A,B) with no gene flow (Fig. 1a), with two sequences sampled per species. Let $g_{00}$, $g_{01}$, $g_{10}$ and $g_{11}$ denote four types of gene trees representing four combinations of two events: whether the two sequences from species *A* coalesce in *A* and whether the two sequences from *B* coalesce in *B*. For example, $g_{01}$ denotes gene trees in which the two sequences from *A* do not coalesce in *A* but the two sequences from *B* coalesce in *B*. The probabilities of the four types of gene trees are given by

(A1)
$$\begin{array}{cc} \;p\left(g_{00}\right) & \;=\;{\text{e}}^{-\left(\frac{2}{\theta_{A}}+\frac{2}{\theta_{B}}\right)\tau_{R}},\\ p\left(g_{01}\right) & \;=\;{\text{e}}^{-\frac{2}{\theta_{A}}\tau_{R}}\left(1-{\text{e}}^{-\frac{2}{\theta_{B}}\tau_{R}}\right),\\ p\left(g_{10}\right) & \;=\;\left(1-{\text{e}}^{-\frac{2}{\theta_{A}}\tau_{R}}\right){\text{e}}^{-\frac{2}{\theta_{B}}\tau_{R}},\\ p\left(g_{11}\right) & \;=\;\left(1-{\text{e}}^{-\frac{2}{\theta_{A}}\tau_{R}}\right)\left(1-{\text{e}}^{-\frac{2}{\theta_{B}}\tau_{R}}\right). \end{array}$$

Let *H* be the height of the gene tree, which is the age of the most recent common ancestor of all four sequences, and let *L* be the tree length, which is the sum of all branch lengths. The expected tree height is

(A2)
$$E\left(H\right)=\sum_{g}E\left(H\;|\;g\right)p\left(g\right),$$

where the summation is over the four gene-tree types and

(A3)
$$\begin{array}{ccc} E\left(H\;|\;g_{00}\right) & = & \tau_{R}+\left(\frac{1}{6}+\frac{1}{3}+1\right)\frac{\theta_{R}}{2}=\tau_{R}+\frac{3}{4}\theta_{R},\\ E\left(H\;|\;g_{01}\right) & = & E\left(H\;|\;g_{10}\right)=\tau_{R}+\left(\frac{1}{3}+1\right)\frac{\theta_{R}}{2}=\tau_{R}+\frac{2}{3}\theta_{R},\\ E\left(H\;|\;g_{11}\right) & = & \tau_{R}+\frac{\theta_{R}}{2}. \end{array}$$

Similarly, the expected tree length is

$$E\left(L\right)=\sum_{g}E\left(L\;|\;g\right)p\left(g\right),$$

with

(A4)
$$\begin{array}{cc} & \;\;\;\;E\left(L\;|\;g_{00}\right)=4\tau_{R}+\left(\frac{1}{3}+\frac{1}{2}+1\right)\theta_{R},\\ & E\left(L\;|\;g_{01}\right)=3\tau_{R}+T_{B}+\left(\frac{1}{2}+1\right)\theta_{R},\\ & E\left(L\;|\;g_{10}\right)=3\tau_{R}+T_{A}+\left(\frac{1}{2}+1\right)\theta_{R},\\ & \;\;\;\;E\left(L\;|\;g_{11}\right)=2\tau_{R}+T_{A}+T_{B}+\theta_{R}, \end{array}$$

where $T_{A}$ (or $T_{B}$) is the averge coalescent time for the two sequences from *A* (or *B*) given that the coalescence occurs in *A* (or *B*), with

(A5)
$$\begin{array}{rl} T_{A} & =E\left(t_{aa}\;|\;t_{aa}<\tau_{R}\right)\\ & =\int_{0}^{\tau_{R}}t\frac{2}{\theta_{A}}{\text{e}}^{-\frac{2}{\theta_{A}}t}\;dt/\left[1-{\text{e}}^{-\frac{2}{\theta_{A}}\tau_{R}}\right]\\ & =\frac{\theta_{A}}{2}-\frac{\tau_{R}}{\exp\;\left\{\frac{2}{\theta_{A}}\tau_{R}\right\}-1}. \end{array}$$

and with $T_{B}$ given similarly.

Following Schierup and Hein (2000), let *S* be the sum of all terminal branch lengths and let *B* be the sum of the two branch lengths around the root. Then $E(S)=\sum_{g}E(S\;|\;g)p(g)$, with

(A6)
$$\begin{array}{cc} & \;E\left(S\;|\;g_{00}\right)=4\tau_{R}+\theta_{R},\\ & E\left(S\;|\;g_{01}\right)=2\tau_{R}+2T_{B}+\frac{2}{3}\theta_{R},\\ & E\left(S\;|\;g_{10}\right)=2\tau_{R}+2T_{A}+\frac{2}{3}\theta_{R},\\ & \;E\left(S\;|\;g_{11}\right)=2T_{A}+2T_{B}, \end{array}$$

and $E(B)=\sum_{g}E(B\;|\;g)p(g)$, with

(A7)
$$\begin{array}{cc} & \;E\left(B\;|\;g_{00}\right)=\frac{1}{3}\left[\left(2+\frac{1}{3}+\frac{1}{6}\right)\frac{\theta_{R}}{2}+\tau_{R}\right]+\frac{1}{6}\left[\left(2+\frac{1}{3}\right)\frac{\theta_{R}}{2}\right],\\ & E\left(B\;|\;g_{01}\right)=\frac{1}{3}\left[\left(2+\frac{1}{3}\right)\frac{\theta_{R}}{2}+\tau_{R}\right]+\frac{1}{6}\left[\left(2+\frac{1}{3}\right)\frac{\theta_{R}}{2}+\left(\tau_{R}-T_{B}\right)\right],\\ & E\left(B\;|\;g_{10}\right)=\frac{1}{3}\left[\left(2+\frac{1}{3}\right)\frac{\theta_{R}}{2}+\tau_{R}\right]+\frac{1}{6}\left[\left(2+\frac{1}{3}\right)\frac{\theta_{R}}{2}+\left(\tau_{R}-T_{A}\right)\right],\\ & \;E\left(B\;|\;g_{11}\right)=\frac{1}{2}\left[\theta_{R}+\left(\tau_{R}-T_{A}\right)+\left(\tau_{R}-T_{B}\right)\right]. \end{array}$$

## Appendix B. Mean and variance of pairwise sequence distances under the MSC, BDI, and UDM models

Let *d* be the pairwise distance between two sequences at a locus, measured as the proportion of variable sites in the sequence, with d=x/n where *x* is the number of variable sites and *n* is the sequence length. Given a coalescent time *t* at this locus, the number of variable sites under the JC mutation model (Jukes and Cantor 1969) has a binomial distribution, given by

(B1)
$$x\;|\;t∼\text{Bin}(n,p),\;p=\frac{3}{4}-\frac{3}{4}{\text{e}}^{-\frac{8}{3}t},$$

with mean *np* and variance np(1−p). Thus the distance *d* has mean

(B2)
$$E(d)=E(\frac{x}{n})=\frac{1}{n}E(E(x\;|\;t))=E(p)$$

and variance

(B3)
$$\begin{array}{rl} V(d) & =V(\frac{x}{n})\\ & =\frac{1}{n^{2}}\left[V(E(x\;|\;t))+E(V(x\;|\;t))\right]\\ & =\frac{1}{n^{2}}\left[V(np)+E(np(1-p))\right]\\ & =V(p)+\frac{1}{n}E(p(1-p)). \end{array}$$

We can approximate E(p), E(p(1−p)) and V(p) using the delta method as follows. Let μ=E(t) and $\sigma^{2}=V(t)$ denote the mean and variance of the coalescent time, and let h=p(1−p). Then

(B4)
$$E(p)\approx p(\mu )+\frac{\sigma^{2}}{2}p^{\prime \prime }(\mu )=p(\mu )-\frac{8}{3}\sigma^{2}{\text{e}}^{-\frac{8}{3}\mu },$$

(B5)
$$\begin{array}{rl} E(p(1-p)) & \approx h(\mu )+\frac{\sigma^{2}}{2}h^{\prime \prime }(\mu )\\ & =h(\mu )+\sigma^{2}\left[\frac{4}{3}{\text{e}}^{-\frac{8}{3}\mu }-8{\text{e}}^{-\frac{16}{3}\mu }\right], \end{array}$$

and

(B6)
$$V(p)\approx \sigma^{2}p^{\prime }(\mu {)}^{2}=4\sigma^{2}{\text{e}}^{-\frac{16}{3}\mu },$$

using $p^{\prime }(\mu )=2{\text{e}}^{-\frac{8}{3}\mu }$, $p^{\prime \prime }(\mu )=-\frac{16}{3}{\text{e}}^{-\frac{8}{3}\mu }$ and $h^{\prime \prime }(\mu )=\frac{8}{3}{\text{e}}^{-\frac{8}{3}\mu }$  $-16{\text{e}}^{-\frac{16}{3}\mu }$.

Next, we provide closed-form expressions of E(t) and V(t) under the BDI (MSC-I) model (Fig. 1b) and the UDM (MSC-M) model (Fig. 1e) for two sequences. Let $t_{aa}$, $t_{ab}$ and $t_{bb}$ denote the coalescent time between two sequences from *A*, between two sequences from *B*, and between one sequence from *A* and one sequence from *B*, respectively. Let the corresponding pairwise sequence distances (or proportions of different sites) be denoted $d_{aa}$, $d_{ab}$ and $d_{bb}$.

Under the BDI model (Fig. 1b), the probability density of $t_{aa}$ is given by

(B7)
$$\begin{array}{r} f_{aa}(t)=\left\{ \begin{array}{cc} \frac{2}{\theta_{A}}{\text{e}}^{-\frac{2}{\theta_{A}}t},\; & \text{if}\;0<t<\tau_{X},\\ {\text{e}}^{-\frac{2}{\theta_{A}}\tau_{X}}[(1-\varphi_{X}{)}^{2}\frac{2}{\theta_{X}}{\text{e}}^{-\frac{2}{\theta_{X}}(t-\tau_{X})}\;\\ +\;\varphi_{X}^{2}\frac{2}{\theta_{Y}}{\text{e}}^{-\frac{2}{\theta_{Y}}(t-\tau_{X})}],\; & \text{if}\;\tau_{X}<t<\tau_{R},\\ {\text{e}}^{-\frac{2}{\theta_{A}}\tau_{X}}[(1-\varphi_{X}{)}^{2}{\text{e}}^{-\frac{2}{\theta_{X}}(\tau_{R}-\tau_{X})}\;\\ +\;\varphi_{X}^{2}{\text{e}}^{-\frac{2}{\theta_{Y}}(\tau_{R}-\tau_{X})}\;\\ +\;2\varphi_{X}(1-\varphi_{X})]\frac{2}{\theta_{R}}{\text{e}}^{-\frac{2}{\theta_{R}}(t-\tau_{R})},\; & \text{if}\;t>\tau_{R}. \end{array}\right. \end{array}$$

The density of $t_{bb}$ is equation (B7) with $\left(\theta_{A},\theta_{X},\theta_{Y},\varphi_{X},\varphi_{Y}\right)$ replaced by $\left(\theta_{B},\theta_{Y},\theta_{X},\varphi_{Y},\varphi_{X}\right)$. Finally, the density of $t_{ab}$ is

(B8)
$$f_{ab}(t)=\left\{ \begin{array}{cc} \varphi_{X}(1-\varphi_{Y})\frac{2}{\theta_{Y}}{\text{e}}^{-\frac{2}{\theta_{Y}}(t-\tau_{X})}\;\\ +\;(1-\varphi_{X})\varphi_{Y}\frac{2}{\theta_{X}}{\text{e}}^{-\frac{2}{\theta_{X}}(t-\tau_{X})},\; & \text{if}\;\tau_{X}<t<\tau_{R},\\ \left[(1-\varphi_{X})(1-\varphi_{Y}\right)\\ +\;\varphi_{X}(1-\varphi_{Y}){\text{e}}^{-\frac{2}{\theta_{Y}}(\tau_{R}-\tau_{X})}\;\\ +\;(1-\varphi_{X})\varphi_{Y}{\text{e}}^{-\frac{2}{\theta_{X}}(\tau_{R}-\tau_{X})}\;\\ +\;\varphi_{X}\varphi_{Y}]\frac{2}{\theta_{R}}{\text{e}}^{-\frac{2}{\theta_{R}}(t-\tau_{R})},\; & \text{if}\;t>\tau_{R}. \end{array}\right.$$

The means of $t_{aa}$ and $t_{ab}$ are given by

(B9)
$$\begin{array}{rl} E(t_{aa}) & =\frac{\theta_{A}}{2}-{\text{e}}^{-\frac{2}{\theta_{A}}\tau_{X}}\left[\tau_{X}+\frac{\theta_{A}}{2}\right]\\ & \;+{\text{e}}^{-\frac{2}{\theta_{A}}\tau_{X}}(1-\varphi_{X}{)}^{2}\{\tau_{X}+\frac{\theta_{X}}{2}\;+\;{\text{e}}^{-\frac{2}{\theta_{X}}(\tau_{R}-\tau_{X})}\left[\frac{\theta_{R}}{2}-\frac{\theta_{X}}{2}\right]\}\\ & \;+{\text{e}}^{-\frac{2}{\theta_{A}}\tau_{X}}\varphi_{X}^{2}\{\tau_{X}+\frac{\theta_{Y}}{2}\;+\;{\text{e}}^{-\frac{2}{\theta_{Y}}(\tau_{R}-\tau_{X})}\left[\frac{\theta_{R}}{2}-\frac{\theta_{Y}}{2}\right]\}\\ & \;+2\varphi_{X}(1-\varphi_{X}){\text{e}}^{-\frac{2}{\theta_{A}}\tau_{X}}\left[\tau_{R}+\frac{\theta_{R}}{2}\right], \end{array}$$

and

(B10)
$$\begin{array}{rl} E(t_{ab}) & =\frac{\theta_{A}}{2}-{\text{e}}^{-\frac{2}{\theta_{A}}\tau_{X}}\left[\tau_{X}+\frac{\theta_{A}}{2}\right]\\ & \;+{\text{e}}^{-\frac{2}{\theta_{A}}\tau_{X}}(1-\varphi_{X}{)}^{2}\{\tau_{X}+\frac{\theta_{X}}{2}\;+\;{\text{e}}^{-\frac{2}{\theta_{X}}(\tau_{R}-\tau_{X})}\left[\frac{\theta_{R}}{2}-\frac{\theta_{X}}{2}\right]\}\\ & \;+{\text{e}}^{-\frac{2}{\theta_{A}}\tau_{X}}\varphi_{X}^{2}\{\tau_{X}+\frac{\theta_{Y}}{2}\;+\;{\text{e}}^{-\frac{2}{\theta_{Y}}(\tau_{R}-\tau_{X})}\left[\frac{\theta_{R}}{2}-\frac{\theta_{Y}}{2}\right]\}\\ & \;+2\varphi_{X}(1-\varphi_{X}){\text{e}}^{-\frac{2}{\theta_{A}}\tau_{X}}\left[\tau_{R}+\frac{\theta_{R}}{2}\right]. \end{array}$$

The variance of $t_{aa}$ is

(B11)
$$V(t_{aa})=E(t_{aa}^{2})-E^{2}(t_{aa}),$$

where

(B12)
$$\begin{array}{rl} E(t_{aa}^{2}) & =\frac{\theta_{A}^{2}}{2}-{\text{e}}^{-\frac{2}{\theta_{A}}\tau_{X}}\left[\tau_{X}^{2}+\tau_{X}\theta_{A}+\frac{\theta_{A}^{2}}{2}\right]\\ & \;+{\text{e}}^{-\frac{2}{\theta_{A}}\tau_{X}}(1-\varphi_{X}{)}^{2}\{\tau_{X}^{2}+\tau_{X}\theta_{X}+\frac{\theta_{X}^{2}}{2}\\ & \;+{\text{e}}^{-\frac{2}{\theta_{X}}(\tau_{R}-\tau_{X})}\left[\tau_{R}\theta_{R}-\tau_{R}\theta_{X}+\frac{\theta_{R}^{2}}{2}-\frac{\theta_{X}^{2}}{2}\right]\}\\ & \;+{\text{e}}^{-\frac{2}{\theta_{A}}\tau_{X}}\varphi_{X}^{2}\{\tau_{X}^{2}+\tau_{X}\theta_{Y}+\frac{\theta_{Y}^{2}}{2}\\ & \;+{\text{e}}^{-\frac{2}{\theta_{Y}}(\tau_{R}-\tau_{X})}\left[\tau_{R}\theta_{R}-\tau_{R}\theta_{Y}+\frac{\theta_{R}^{2}}{2}-\frac{\theta_{Y}^{2}}{2}\right]\}\\ & \;+2\varphi_{X}(1-\varphi_{X}){\text{e}}^{-\frac{2}{\theta_{A}}\tau_{X}}\left[\tau_{R}^{2}+\tau_{R}\theta_{R}+\frac{\theta_{R}^{2}}{2}\right]. \end{array}$$

The variance of $t_{ab}$ is $V(t_{ab})=E(t_{ab}^{2})-E^{2}(t_{ab})$ with

(B13)
$$\begin{array}{rl} E(t_{ab}^{2}) & =\varphi_{X}(1-\varphi_{Y})\{\tau_{X}^{2}+\tau_{X}\theta_{Y}+\frac{\theta_{Y}^{2}}{2}\\ & \;+\;{\text{e}}^{-\frac{2}{\theta_{Y}}(\tau_{R}-\tau_{X})}\left[\tau_{R}\theta_{R}-\tau_{R}\theta_{Y}+\frac{\theta_{R}^{2}}{2}-\frac{\theta_{Y}^{2}}{2}\right]\}\\ & \;\;+(1-\varphi_{X})\varphi_{Y}\{\tau_{X}^{2}+\tau_{X}\theta_{X}+\frac{\theta_{X}^{2}}{2}\\ & \;+\;{\text{e}}^{-\frac{2}{\theta_{X}}(\tau_{R}-\tau_{X})}\left[\tau_{R}\theta_{R}-\tau_{R}\theta_{X}+\frac{\theta_{R}^{2}}{2}-\frac{\theta_{X}^{2}}{2}\right]\}\\ & \;\;+\left[(1-\varphi_{X})(1-\varphi_{Y})+\varphi_{X}\varphi_{Y}\right]\left[\tau_{R}^{2}+\tau_{R}\theta_{R}+\frac{\theta_{R}^{2}}{2}\right]. \end{array}$$

The mean and variance of $t_{bb}$ is given by equations (B9) and (B11) with $\left\{\theta_{A},\theta_{X},\theta_{Y},\varphi_{X},\varphi_{Y}\right\}$ replaced by $\left\{\theta_{B},\theta_{Y},\theta_{X},\varphi_{Y},\varphi_{X}\right\}$.

Under the UDM model (Fig. 1e), the probability densities of $t_{aa}$, $t_{ab}$ and $t_{bb}$ are given in Thawornwattana et al. (2025, Eqns. S8–S10, with $\tau_{T}=0$). The first two moment are as follows:

(B14)
$$\begin{array}{rl} E(t_{aa}) & =\frac{\theta_{A}}{2}-{\text{e}}^{-\frac{2}{\theta_{A}}\tau_{R}}\left(\tau_{R}+\frac{\theta_{A}}{2}\right)+{\text{e}}^{-\frac{2}{\theta_{A}}\tau_{R}}\left(\tau R+\frac{\theta_{R}}{2}\right)\\ E(t_{aa}^{2}) & =\frac{\theta_{A}}{2}-{\text{e}}^{-\frac{2}{\theta_{A}}\tau_{R}}\left(\tau_{R}^{2}+\tau_{R}\theta_{A}+\frac{\theta_{A}^{2}}{2}\right)\\ & \;+{\text{e}}^{-\frac{2}{\theta_{A}}\tau_{R}}\left(\tau R^{2}+\tau_{R}\theta_{R}+\frac{\theta_{R}^{2}}{2}\right), \end{array}$$

(B15)
$$\begin{array}{rl} E(t_{ab}) & =\frac{2\varpi_{AB}}{2-\theta_{A}\varpi_{AB}}\left[I_{1}(\varpi_{AB},\tau_{R})-I_{1}(\frac{2}{\theta_{A}},\tau_{R})\right]\\ & \;+\left[\frac{2{\text{e}}^{-\varpi_{AB}\tau_{R}}}{2-\theta_{A}\varpi_{AB}}-\frac{\theta_{A}\varpi_{AB}{\text{e}}^{-\frac{2}{\theta_{A}}\tau_{R}}}{2-\theta_{A}\varpi_{AB}}\right]\frac{2}{\theta_{R}}J_{1}(\frac{2}{\theta_{R}},\tau_{R})\\ E(t_{ab}^{2}) & =\frac{2\varpi_{AB}}{2-\theta_{A}\varpi_{AB}}\left[I_{2}(\varpi_{AB},\tau_{R})-I_{2}(\frac{2}{\theta_{A}},\tau_{R})\right]\\ & \;+\left[\frac{2{\text{e}}^{-\varpi_{AB}\tau_{R}}}{2-\theta_{A}\varpi_{AB}}-\frac{\theta_{A}\varpi_{AB}{\text{e}}^{-\frac{2}{\theta_{A}}\tau_{R}}}{2-\theta_{A}\varpi_{AB}}\right]\frac{2}{\theta_{R}}J_{2}(\frac{2}{\theta_{R}},\tau_{R}) \end{array}$$

and

(B16)
$$\begin{array}{rl} E(t_{bb}) & =\frac{2}{\theta_{A}}H_{1}(\tau_{R})+\frac{2}{\theta_{B}}I_{1}(\frac{2}{\theta_{B}}+2\varpi_{AB},\tau_{R})\\ & \;+\left[1-g(\tau_{R})\right]\frac{2}{\theta_{R}}J_{1}(\frac{2}{\theta_{R}},\tau_{R})\\ E(t_{bb}^{2}) & =\frac{2}{\theta_{A}}H_{2}(\tau_{R})+\frac{2}{\theta_{B}}I_{2}(\frac{2}{\theta_{B}}+2\varpi_{AB},\tau_{R})\\ & \;+\left[1-g(\tau_{R})\right]\frac{2}{\theta_{R}}J_{2}(\frac{2}{\theta_{R}},\tau_{R}), \end{array}$$

where

(B17)
$$\begin{array}{c} I_{1}(a,t)=\int_{0}^{\tau_{R}}t{\text{e}}^{-at}\;dt=\frac{1}{a^{2}}\left[1-{\text{e}}^{-a\tau_{R}}(1+a\tau_{R})\right],\\ I_{2}(a,t)=\int_{0}^{\tau_{R}}t^{2}{\text{e}}^{-at}\;dt=\frac{1}{a^{3}}\left[2-{\text{e}}^{-a\tau_{R}}(2+a\tau (2+a\tau_{R}))\right],\\ J_{1}(a,t)=\int_{\tau_{R}}^{\infty }t{\text{e}}^{-a(t-\tau_{R})}\;dt=\frac{1}{a^{2}}(1+a\tau_{R}),\\ J_{2}(a,t)=\int_{\tau_{R}}^{\infty }t^{2}{\text{e}}^{-a(t-\tau_{R})}\;dt=\frac{1}{a^{3}}\left[2+a\tau_{R}(2+a\tau_{R})\right],\\ H_{1}(t)=\int_{0}^{\tau_{R}}t\;h(t)\;dt,\\ H_{2}(t)=\int_{0}^{\tau_{R}}t^{2}\;h(t)\;dt, \end{array}$$

with

(B18)
h(t)=ϖAB2θAθB(2−ϖABθA)(2+ϖABθB)(θA−θB+ϖABθAθB)×[(2−ϖABθA)θBe−(2θB+2ϖAB)t−θA(2+ϖABθB)e−2θAt+2(θA−θB+ϖABθAθB)e−ϖABt2θB]

and

(B19)
$$\begin{array}{rl} g(t) & =1-h(t)-\frac{2\theta_{B}\varpi_{AB}}{2+\theta_{B}\varpi_{AB}}\left[{\text{e}}^{-\varpi_{AB}t}-{\text{e}}^{-(\frac{2}{\theta_{B}}+2\varpi_{AB})t}\right]\\ & \;-{\text{e}}^{-(\frac{2}{\theta_{B}}+2\varpi_{AB})t}. \end{array}$$

Singularities of these quantities at $\varpi_{AB}=0$ and $\varpi_{AB}=\frac{2}{\theta_{A}}$ are resolved by taking an appropriate limit.

## Appendix C. Likelihood ratio test using integrated likelihood

### Example C1. One-sample test

Consider the one-sample test with unknown variance. The null hypothesis is $H_{0}\;:\;N(0,\sigma^{2})$ with the parameter vector $\theta_{0}=(\sigma^{2})$, and the alternative hypothesis is $H_{1}\;:\;N(\mu ,\sigma^{2})$, with $\theta =(\mu ,\sigma^{2})$. The data are an i.i.d. sample of size *n*, $\left(x_{i}\right)$, summarized as the sample mean $\bar{x}$ and variance $s^{2}=\frac{1}{n}\sum (x_{i}-\bar{x}{)}^{2}$, with $x=(\bar{x},s^{2})$. The likelihood functions under the two models are

(C1)
$$\begin{array}{c} L_{0}(\theta_{0})=(2\pi \sigma^{2}{)}^{-n/2}\exp\;\left\{-\frac{1}{2\sigma^{2}}[n{\bar{x}}^{2}+ns^{2}]\right\},\\ L_{1}(\theta )=(2\pi \sigma^{2}{)}^{-n/2}\exp\;\left\{-\frac{1}{2\sigma^{2}}[n(\bar{x}-\mu {)}^{2}+ns^{2}]\right\}. \end{array}$$

(a) To conduct a Bayesian test, we specify an inverse gamma prior for the nuisance parameter, $\sigma^{2}∼$ IG(α,β) under $H_{0}$. Under $H_{1}$, we assign $\sigma^{2}∼$ IG(α,β) and then $\mu \;|\;\sigma^{2}∼N(0,k\sigma^{2})$ with *k* given, so that the joint prior is

(C2)
$$p(\theta )=\frac{\beta^{\alpha }}{\Gamma (\alpha )}(\sigma^{2}{)}^{-\alpha -1}{\text{e}}^{-\beta /\sigma^{2}}\times \frac{1}{\sqrt{2\pi k\sigma^{2}}}{\text{e}}^{-\frac{1}{2k\sigma^{2}}\mu^{2}}.$$

The marginal likelihood values under $H_{0}$ and $H_{1}$ are

(C3)
$$\begin{array}{c} m_{0}=(2\beta {)}^{\alpha }\frac{\Gamma (\alpha +\frac{n}{2})}{\Gamma (\alpha )}\pi^{-\frac{n}{2}}{\left[2\beta +ns^{2}+n{\bar{x}}^{2}\right]}^{-\alpha -\frac{n}{2}},\\ m_{1}=(2\beta {)}^{\alpha }\frac{\Gamma (\alpha +\frac{n}{2})}{\Gamma (\alpha )}(1+nk{)}^{-\frac{1}{2}}\pi^{-\frac{n}{2}}{\left[2\beta +ns^{2}+\frac{n{\bar{x}}^{2}}{1+nk}\right]}^{-\alpha -\frac{n}{2}} \end{array}$$

(O’Hagan and Forster 2004, p. 170).

The Bayes factor is

(C4)
$$B_{10}=\frac{m_{1}}{m_{0}}=\frac{1}{\sqrt{1+nk}}{\left[\frac{2\beta +ns^{2}+n{\bar{x}}^{2}}{2\beta +ns^{2}+\frac{n{\bar{x}}^{2}}{1+nk}}\right]}^{\alpha +\frac{n}{2}}.$$

(O’Hagan and Forster 2004, eq. 7.10).

(b) To conduct the LRT, note that under $H_{0}$, the MLE is ${\hat{\sigma }}^{2}={\bar{x}}^{2}+s^{2}$ and $L_{0}=[2\pi \text{e}({\bar{x}}^{2}+s^{2}){]}^{-n/2}$. Under $H_{1}$, the MLE is $\left(\hat{\mu },{\hat{\sigma }}^{2})=(\bar{x},s^{2}\right)$ and $L_{1}=(2\pi \text{e}s^{2}{)}^{-n/2}$. The LRT statistic is

(C5)
$$\Delta =2\log\;\frac{L_{1}}{L_{0}}=n\log\;\left[1+\frac{{\bar{x}}^{2}}{s^{2}}\right].$$

Under $H_{0}$, $\Delta \approx t^{2}$ where $t=\frac{\bar{x}}{\sqrt{s^{2}/(n-1)}}∼$  $t_{n-1}$ so that Δ is asymptotically $\chi_{1}^{2}$ when n→∞.

(c) To use integrated likelihood, we have $L_{0}^{*}=m_{0}$, and

(C6)
$$\begin{array}{rl} L_{1}^{*}(\mu ) & =\int p(\sigma^{2})L_{1}(\mu ,\sigma^{2})\;d\sigma^{2}\\ & =(2\beta {)}^{\alpha }\frac{\Gamma (\alpha +\frac{n}{2})}{\Gamma (\alpha )}\pi^{-\frac{n}{2}}{\left[2\beta +ns^{2}+n(\bar{x}-\mu {)}^{2}\right]}^{-\alpha -\frac{n}{2}}, \end{array}$$

This is maximized at ${\hat{\mu }}^{*}=\bar{x}$. The integrated likelihood ratio test statistic is

(C7)
$$\begin{array}{rl} \Delta^{*} & =2\log\;\frac{L_{1}^{*}({\hat{\mu }}^{*})}{L_{0}^{*}}\\ & =(2\alpha +n)\log\;\frac{2\beta +ns^{2}+n{\bar{x}}^{2}}{2\beta +ns^{2}}\\ & =(2\alpha +n)\log\;\left[1+\frac{n{\bar{x}}^{2}}{2\beta +ns^{2}}\right]. \end{array}$$

This becomes equation (C5) for the extreme prior α=0,β=0.

### Example C2. Two-sample test

Consider a test of the equality of two population means with the variance given. The null hypothesis is $H_{0}\;:\;\mu_{1}=\mu_{2}=\mu$ while the alternative hypothesis is $H_{1}\;:\;\mu_{1}\neq \mu_{2}$. The data are samples taken from the two populations, $\left(x_{ij}\right)$, i=1,2; j=1,…,n. The likelihood model specifies $x_{ij}∼N(\mu ,1)$ under $H_{0}$ and $x_{ij}∼N(\mu_{i},1)$ under $H_{1}$. The data are summarized as the sample means, $x=({\bar{x}}_{1},{\bar{x}}_{2})$, with ${\bar{x}}_{i}=\frac{1}{n}\sum_{j}x_{ij}$, i=1,2.

(a) In a Bayesian analysis, we assign the prior μ∼N(0,1/(2τ)) under $H_{0}$, and the prior

(C8)
$$\begin{array}{rl} \;p(\mu_{1},\mu_{2}) & =\phi (\mu_{1};0,1/\tau )\phi (\mu_{2};0,1/\tau )\\ & =\frac{\tau }{2\pi }\exp\;\left\{-\frac{\tau }{2}\left(\mu_{1}^{2}+\mu_{2}^{2}\right)\right\} \end{array}$$

under $H_{1}$, with *τ* given.

The posterior of parameters under $H_{1}$ is

(C9)
$$p(\mu_{1},\mu_{2}|x)=\phi \left(\mu_{1};\frac{n{\bar{x}}_{1}}{\tau +n},\frac{1}{\tau +n}\right)\phi \left(\mu_{2};\frac{n{\bar{x}}_{2}}{\tau +n},\frac{1}{\tau +n}\right).$$

Under $H_{0}$, the marginal likelihood is

(C10)
$$\begin{array}{rl} m_{0} & =p({\bar{x}}_{1},{\bar{x}}_{2}|H_{0})\\ & =\int \phi \left(\mu ;0,\frac{1}{2\tau }\right)\phi \left({\bar{x}}_{1};\mu ,\frac{1}{n}\right)\phi \left({\bar{x}}_{2};\mu ,\frac{1}{n}\right)\;d\mu \\ & =\frac{n}{2\pi }\sqrt{\frac{\tau }{\tau +n}}\exp\;\left\{\frac{n^{2}({\bar{x}}_{1}+{\bar{x}}_{2}{)}^{2}}{4(\tau +n)}-\frac{n({\bar{x}}_{1}^{2}+{\bar{x}}_{2}^{2})}{2}\right\}\\ & =\frac{n}{2\pi }\sqrt{\frac{\tau }{\tau +n}}\exp\;\left\{\frac{-n^{2}({\bar{x}}_{1}-{\bar{x}}_{2}{)}^{2}-2\tau n({\bar{x}}_{1}^{2}+{\bar{x}}_{2}^{2})}{4(\tau +n)}\right\}. \end{array}$$

Under $H_{1}$, the marginal likelihood is

(C11)
$$\begin{array}{rl} m_{1} & =p({\bar{x}}_{1},{\bar{x}}_{2})\\ & =\phi \left({\bar{x}}_{1};0,\frac{1}{\tau }+\frac{1}{n}\right)\cdot \phi \left({\bar{x}}_{2};0,\frac{1}{\tau }+\frac{1}{n}\right)\\ & =\frac{\tau n}{2\pi (\tau +n)}\exp\;\left\{-\frac{\tau n}{2(\tau +n)}({\bar{x}}_{1}^{2}+{\bar{x}}_{2}^{2})\right\}. \end{array}$$

Thus the Bayes factor is

(C12)
$$B_{10}=\frac{m_{1}}{m_{0}}=\sqrt{\frac{\tau }{\tau +n}}\exp\;\left\{\frac{n^{2}({\bar{x}}_{1}-{\bar{x}}_{2}{)}^{2}}{4(\tau +n)}\right\}.$$

(b) To conduct the LRT, note that under $H_{0}$, the MLE is $\hat{\mu }=({\bar{x}}_{1}+{\bar{x}}_{2})/2$ and $L_{0}=\phi (\hat{\mu };{\bar{x}}_{1},\frac{1}{n})\phi (\hat{\mu };{\bar{x}}_{2},\frac{1}{n})$. Under $H_{1}$, the MLE is $\left({\hat{\mu }}_{1},{\hat{\mu }}_{2})=({\bar{x}}_{1},{\bar{x}}_{2}\right)$ and $L_{1}=\phi ({\bar{x}}_{1};{\bar{x}}_{1},\frac{1}{n})\phi ({\bar{x}}_{2};{\bar{x}}_{2},\frac{1}{n})$  $=\frac{n}{2\pi }$. The LRT statistic is

(C13)
$$\Delta =2\log\;\frac{L_{1}}{L_{0}}=\frac{|{\bar{x}}_{1}-{\bar{x}}_{2}{|}^{2}}{2/n}=z^{2},$$

which has the $\chi_{1}^{2}$ distribution as $z=\frac{{\bar{x}}_{1}-{\bar{x}}_{2}}{\sqrt{2/n}}∼N(0,1)$ under $H_{0}$. In other words the LRT is equivalent to the *z*-test. Note that here the $\chi_{1}^{2}$ null distribution is exact and does not require a large *n*. Note that the MLE of $\delta =\mu_{2}-\mu_{1}$ is $\hat{\delta }={\bar{x}}_{2}-{\bar{x}}_{1}$.

(c) To use integrated likelihood, re-parametrize $H_{1}$ so that $\theta^{\prime }=(\mu ,\delta )=(\mu_{1},\mu_{2}-\mu_{1})$. Suppose we use μ∼N(0,1/(2τ)) under both $H_{0}$ and $H_{1}$. We have $L_{0}^{*}=m_{0}$, and

(C14)
$$\begin{array}{rl} L_{1}^{*}(\delta ) & =\int p(\mu_{1})\phi ({\bar{x}}_{1};\mu_{1},\frac{1}{n})\phi ({\bar{x}}_{2};\mu_{1}+\delta ,\frac{1}{n})\;d\mu_{1}\\ & =\frac{n}{2\pi }\sqrt{\frac{\tau }{\tau +n}}\\ & \;\times \exp\;\{-[\delta^{2}(n^{2}+2n\tau )+\delta [2n^{2}({\bar{x}}_{1}-{\bar{x}}_{2})-4n\tau {\bar{x}}_{2}]\\ & \;+n^{2}({\bar{x}}_{1}-{\bar{x}}_{2}{)}^{2}+2n\tau ({\bar{x}}_{1}^{2}+{\bar{x}}_{2}^{2})]/[4(\tau +n)]\}. \end{array}$$

The integrated likelihood ratio is

(C15)
$$\begin{array}{rl} R^{*}(\delta ) & =L_{1}^{*}(\delta )/L_{0}^{*}\\ & =\exp\;\left\{-\frac{\delta^{2}(n^{2}+2n\tau )-\delta [(2n^{2}({\bar{x}}_{2}-{\bar{x}}_{1})+4n\tau {\bar{x}}_{2}]}{4(n+\tau )}\right\}. \end{array}$$

Note that if a>0, the function ${\text{e}}^{-(ay^{2}+by)}$ has the maximum ${\text{e}}^{b^{2}/(4a)}$ at y=b/(2a). $R^{*}(\delta )$ and thus $L_{1}^{*}(\delta )$ are maximized at

(C16)
$${\hat{\delta }}^{*}=\frac{n({\bar{x}}_{2}-{\bar{x}}_{1})+2\tau {\bar{x}}_{2}}{n+2\tau }.$$

This is a weighted average of $\left({\bar{x}}_{2}-{\bar{x}}_{1}\right)$ and ${\bar{x}}_{2}$, with weights being the sample precision *n* and the prior precision 2τ.

Thus the integrated likelihood ratio test statistic is

(C17)
$$\begin{array}{rl} \Delta^{*} & =2\log\;R^{*}({\hat{\delta }}^{*})=\frac{{\left[2n^{2}({\bar{x}}_{2}-{\bar{x}}_{1})+4n\tau {\bar{x}}_{2}\right]}^{2}}{2(n^{2}+2n\tau )\cdot 4(\tau +n)}\\ & =\frac{{\left[({\bar{x}}_{2}-{\bar{x}}_{1})+2\tau /n\cdot {\bar{x}}_{2}\right]}^{2}}{2/n\cdot (1+2\tau /n)(1+\tau /n)}. \end{array}$$

$\Delta^{*}$ becomes Δ of equation (C13) if τ=0 but otherwise does not have the $\chi_{1}^{2}$ distribution. If the prior precision relative to the sample size, τ/n, is large and the prior mean 0 differs from the sample mean ${\bar{x}}_{2}$, the statistic $\Delta^{*}$ may differ considerably from Δ.

## Data Availability

Simulated datasets and scripts for simulation and Bpp analysis are available on Zenodo at https://doi.org/10.5281/zenodo.17019348.
