# Supplementary material for: On the robustness of Bayesian inference of gene flow to intragenic recombination and natural selection
Source: Mol Biol Evol. 2025 Dec 12;43(1):msaf327. doi: 10.1093/molbev/msaf327 (PMC12759005; doi:10.1093/molbev/msaf327)
Supplement: msaf327_Supplementary_Data [file msaf327_supplementary_data.pdf]

Supplemental Information

**On the robustness of Bayesian inference of gene flow to intragenic recombination and natural selection**

Thawornwattana, Y., Rannala, B., and Yang Z.

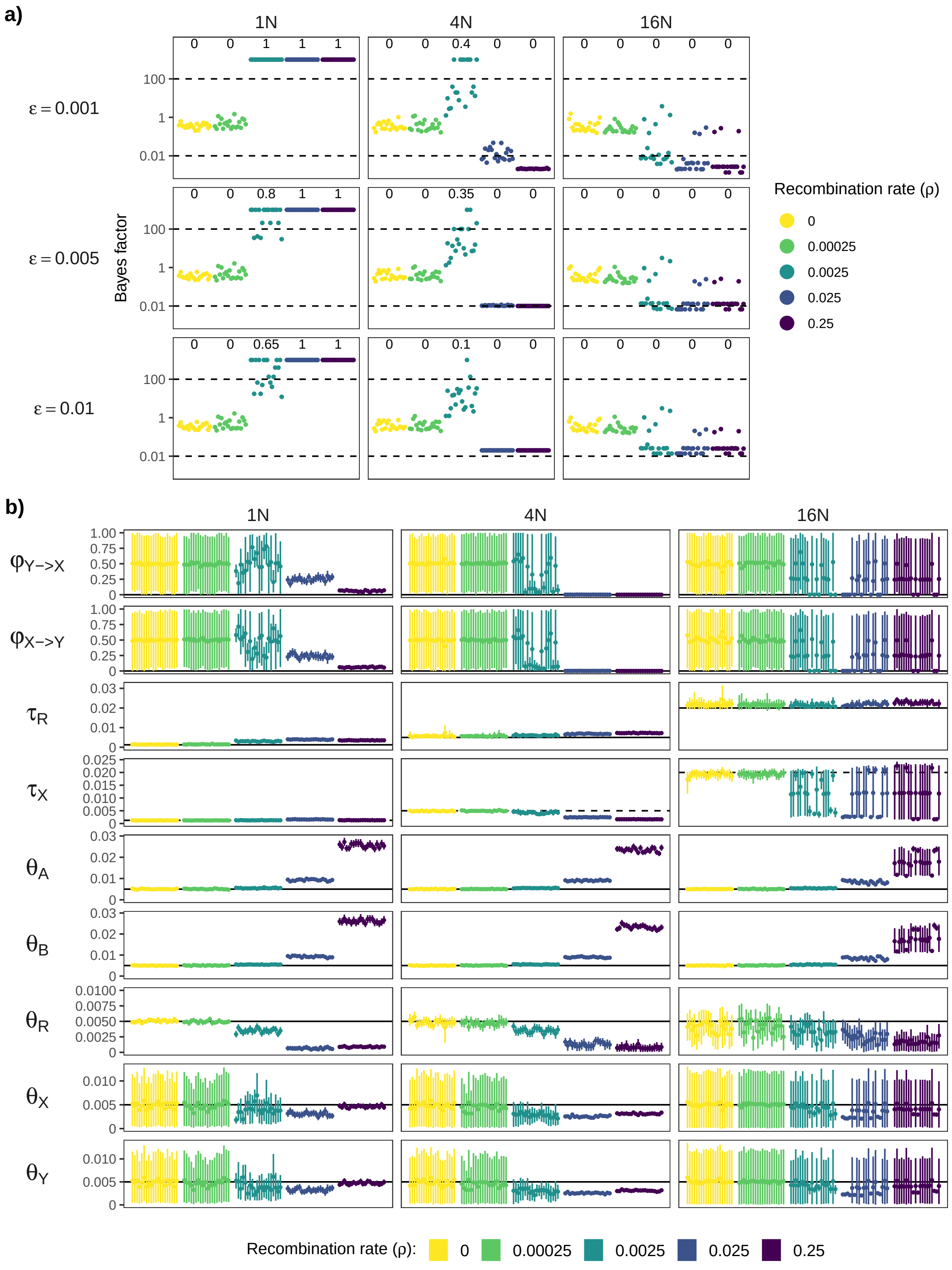


**Fig. S1**: **a)** Bayes factors and false positive rates for testing gene flow under the BDI model (fig. 1**b)** for data simulated under neutral evolution with different recombination rates $\rho = 4Nr$ (per site). Rows are different cutoff values used in the S-D approach ($\varepsilon_\varphi = \varepsilon$ and $\varepsilon_\tau = \varepsilon\tau$). Horizontal dashed lines indicate two significance thresholds at $B_{10} = 100$ (gene flow) and 0.01 (no gene flow). Values larger than 1000 are shown as 1000. Numbers at the top of each subplot are false positive rates from 20 replicates. **b)** Parameter estimates (dot for posterior mean and bar for 95% HPD CI) for data of panel **a**. Horizontal solid lines indicate the true value for $\tau_R$, $\theta_A$, $\theta_B$ as well as $\theta_X$ and $\theta_Y$. The horizontal dashed line for $\tau_X$ indicates true $\tau_R$.

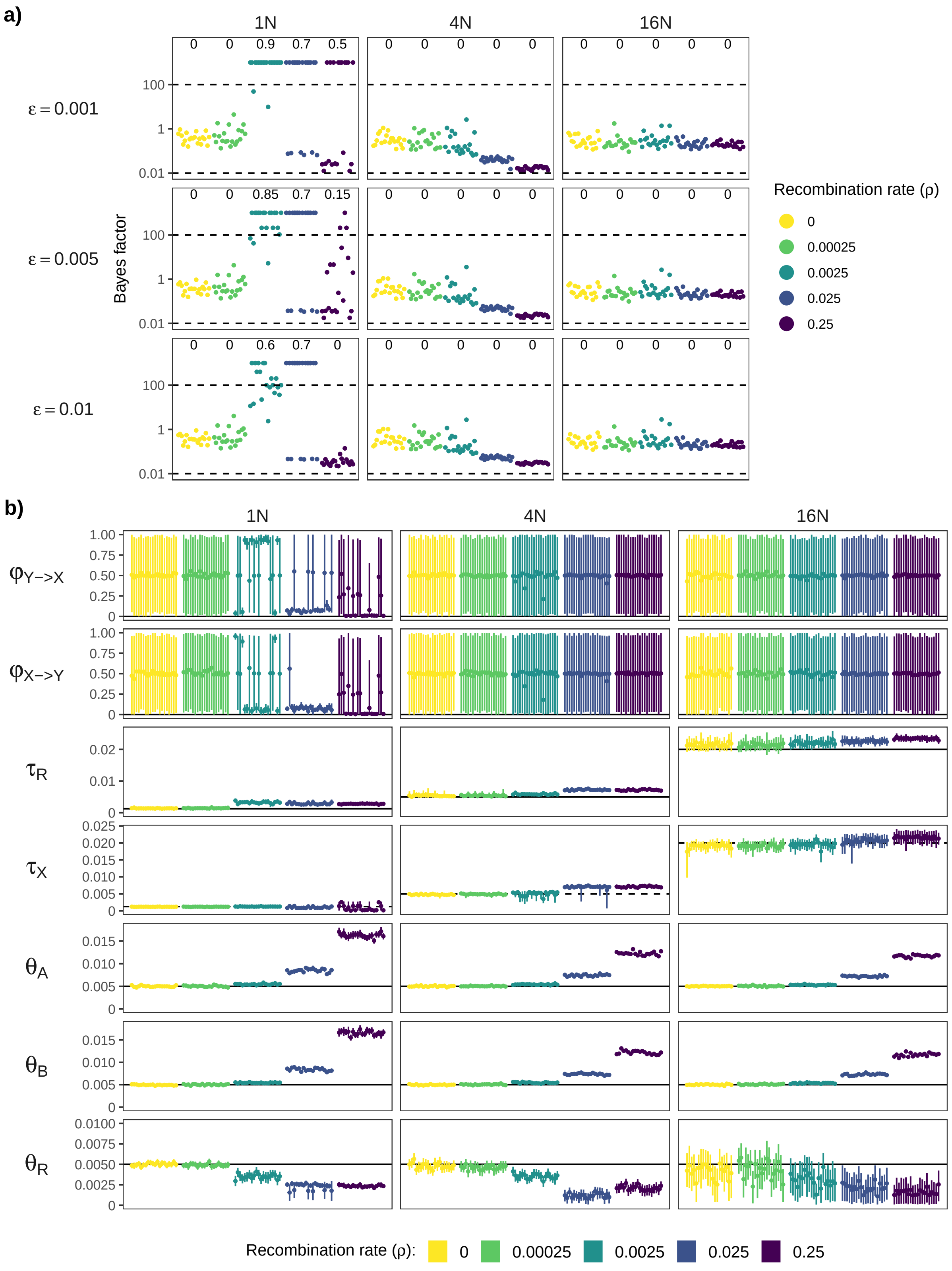


**Fig. S2**: **a)** Bayes factors and false positive rates under the BDI model (fig. 1**b**), assuming $\theta_X = \theta_A$ and $\theta_Y = \theta_B$, for data simulated under neutral evolution with different recombination rates. **b)** Parameter estimates for data of panel **a**. See legend to Figure S1.

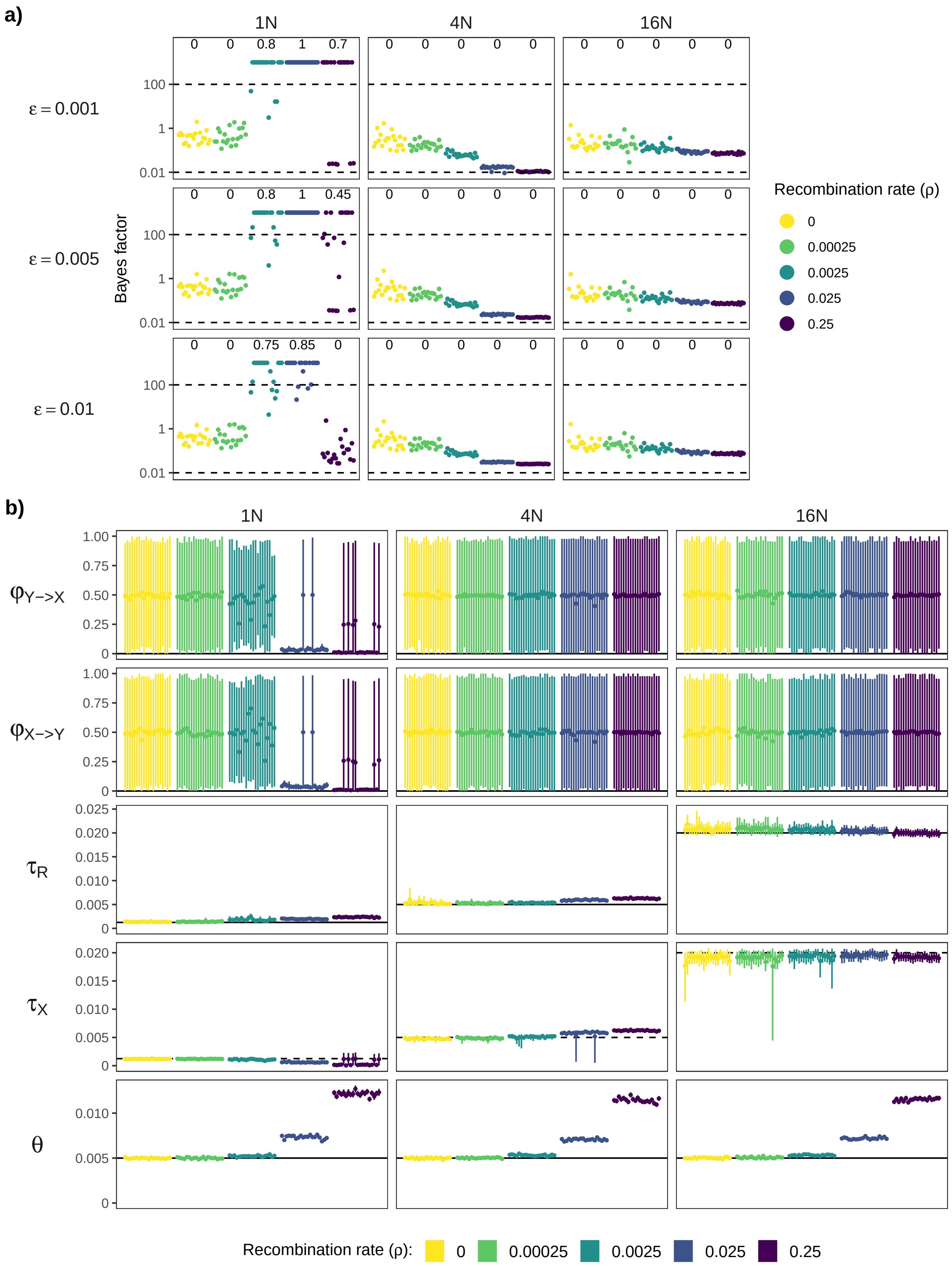


**Fig. S3**: **a)** Bayes factors and false positive rates using the BDI model (fig. 1**b**), assuming all populations have the same size ($\theta$), for data simulated under neutral evolution with different recombination rates. **b)** Parameter estimates for data of panel **a**. See legend to Figure S1.

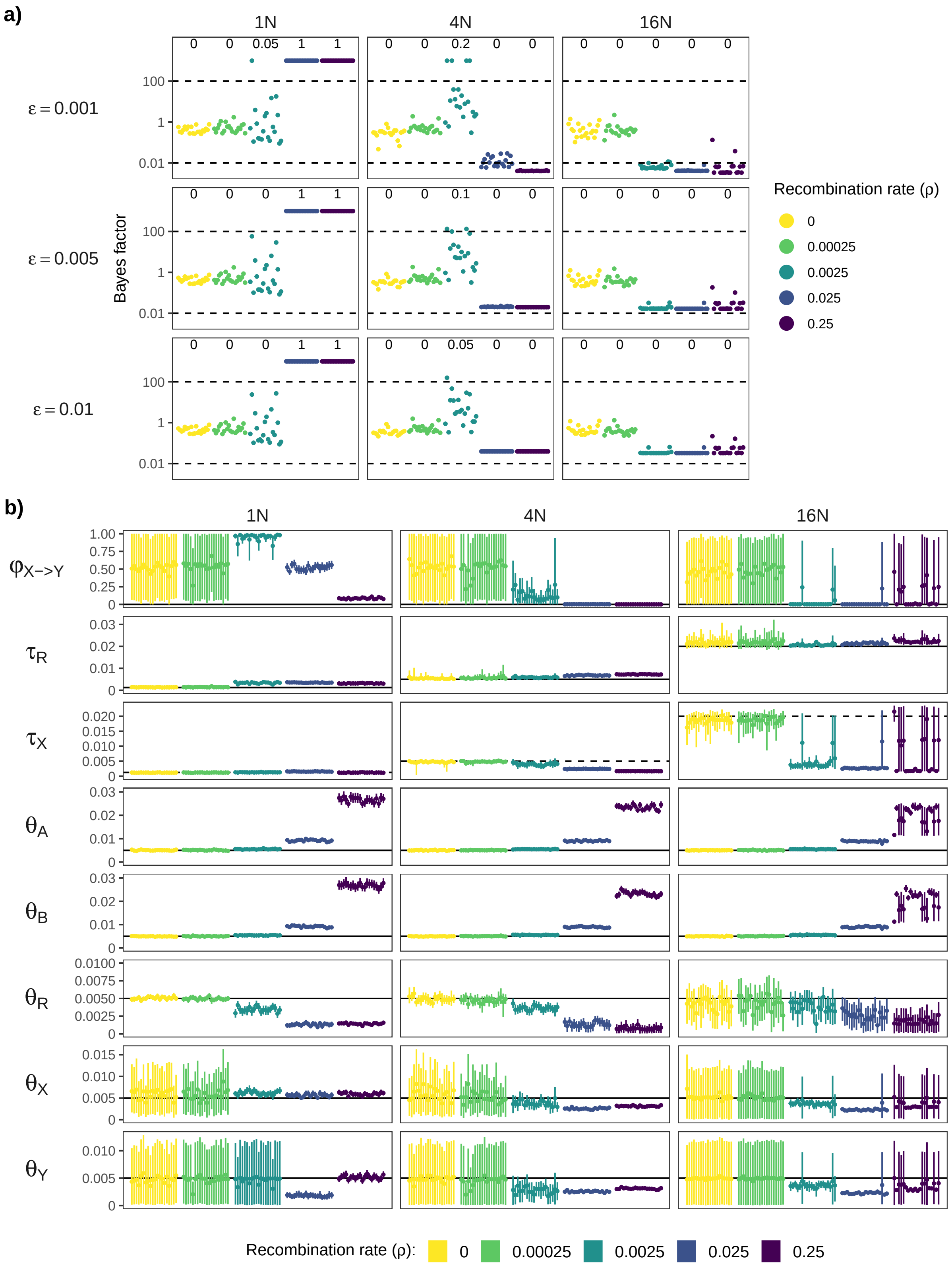


**Fig. S4**: **a)** Bayes factors and false positive rates under the UDI model (fig. 1**c**), for data simulated under neutral evolution with different recombination rates. **b)** Parameter estimates for data of panel **a**. See legend to Figure S1.

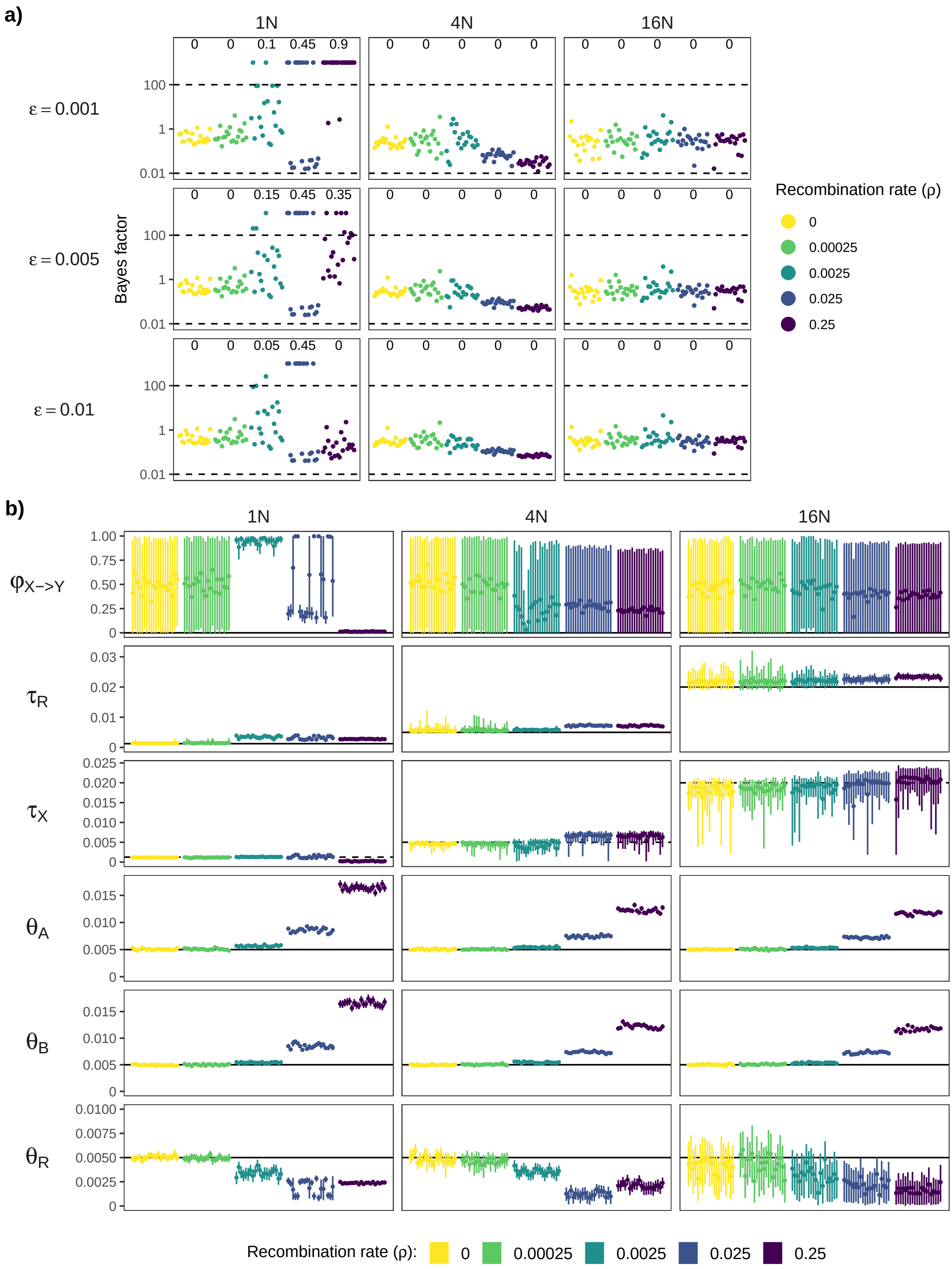


**Fig. S5**: **a)** Bayes factors and false positive rates under the UDI model (fig. 1**c**), assuming $\theta_X = \theta_A$ and $\theta_Y = \theta_B$, for data simulated under neutral evolution with different recombination rates. **b)** Parameter estimates for data of panel **a**. See legend to Figure S1.

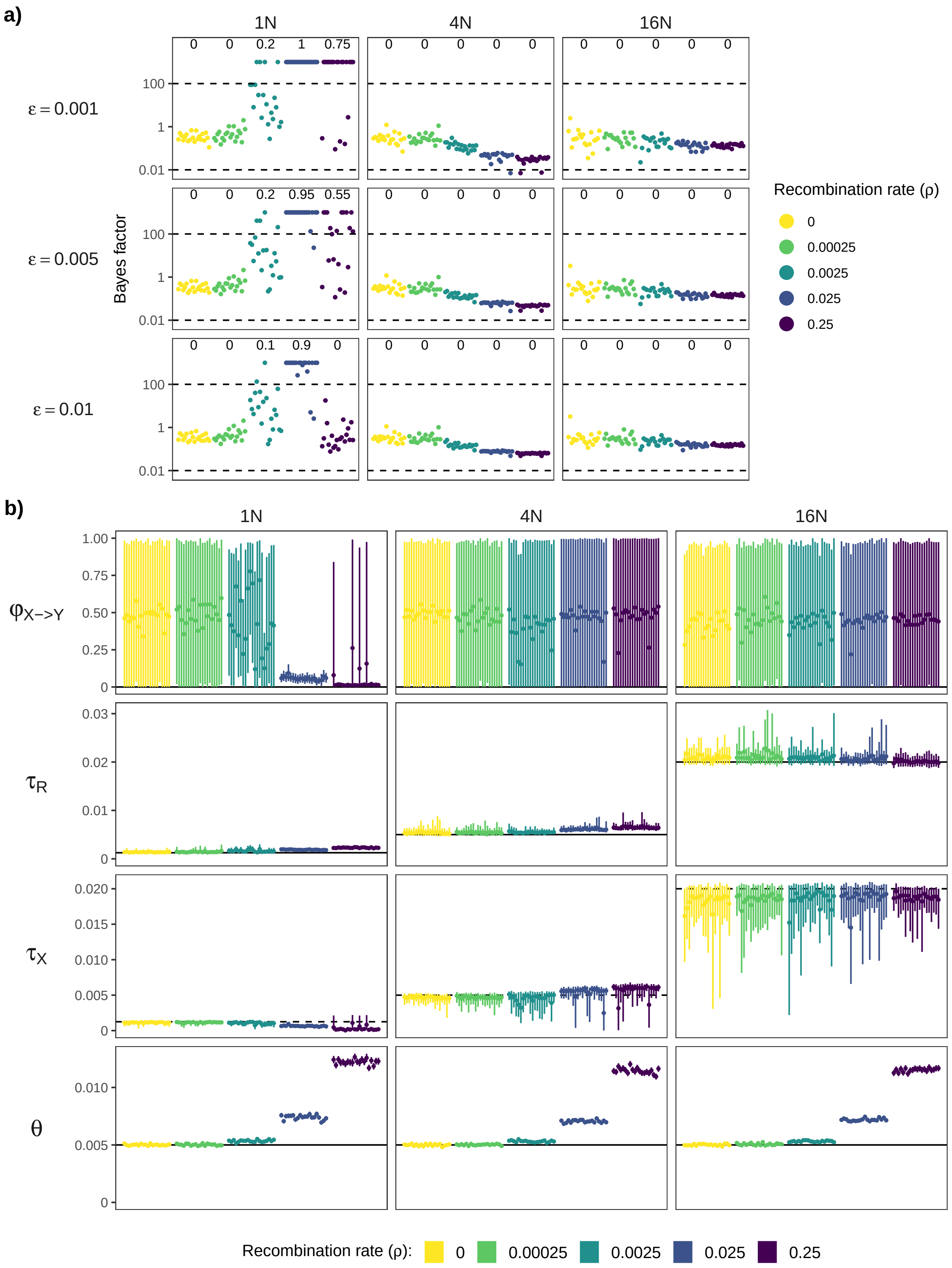


**Fig. S6**: **a)** Bayes factors and false positive rates under the UDI model (fig. 1**c**), assuming all populations have the same size ($\theta$), for data simulated under neutral evolution with different recombination rates. **b)** Parameter estimates for data of panel **a**. See legend to Figure S1.

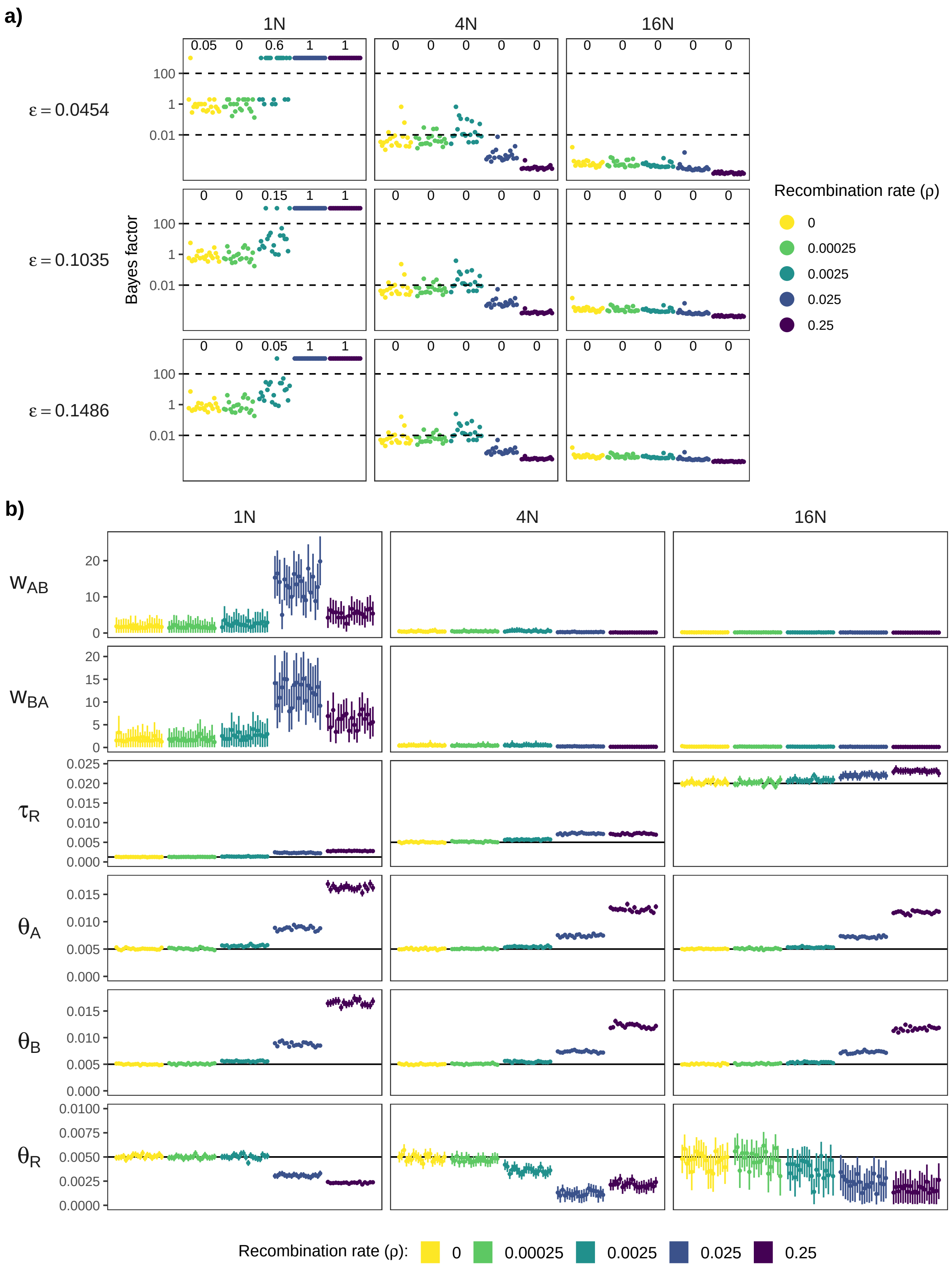


**Fig. S7**: **a)** Bayes factors and false positive rates under the BDM model (fig. 1**d**), for data simulated under neutral evolution with different recombination rates. **b)** Parameter estimates for data of panel **a**. See legend to Figure S1.

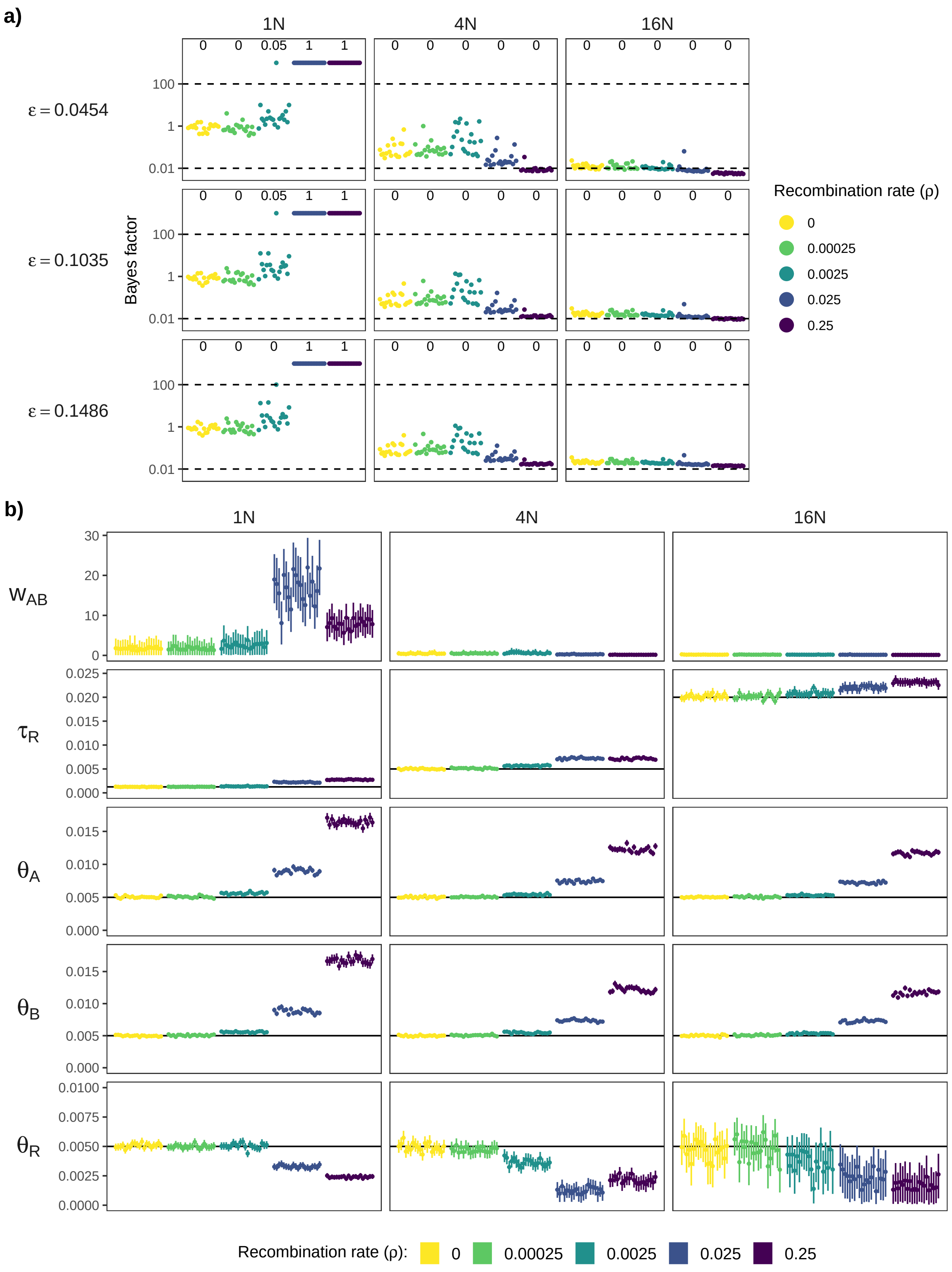


**Fig. S8**: **a)** Bayes factors and false positive rates under the UDM model (fig. 1**e**), for data simulated under neutral evolution with different recombination rates. **b)** Parameter estimates for data of panel **a**. See legend to Figure S1.

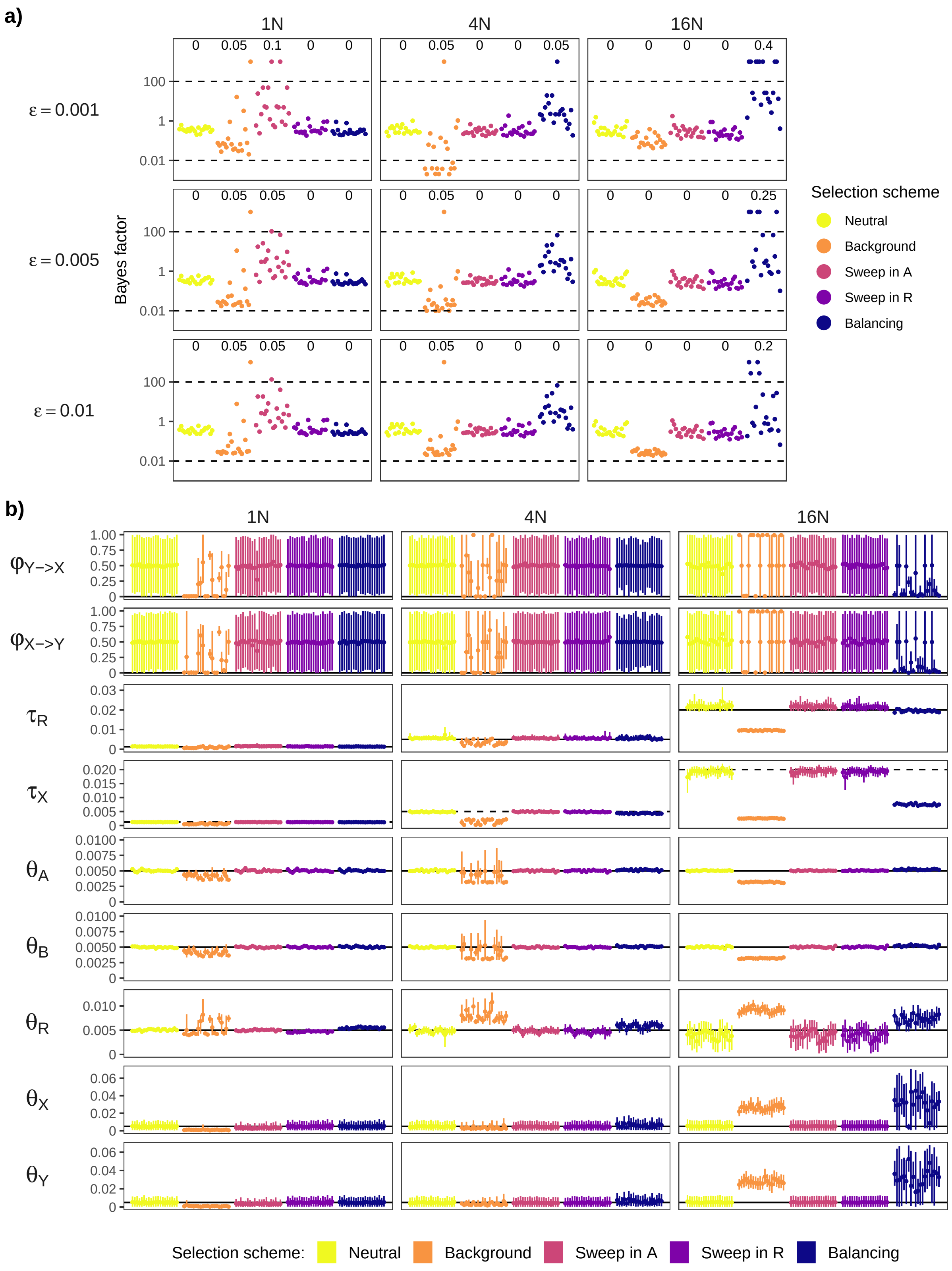


**Fig. S9**: **a)** Bayes factors and false positive rates using the BDI model (fig. 1**b**), for data simulated under different selection schemes with no recombination ($\rho = 0$). **b)** Parameter estimates for data of panel **a**. See legend to Figure S1.

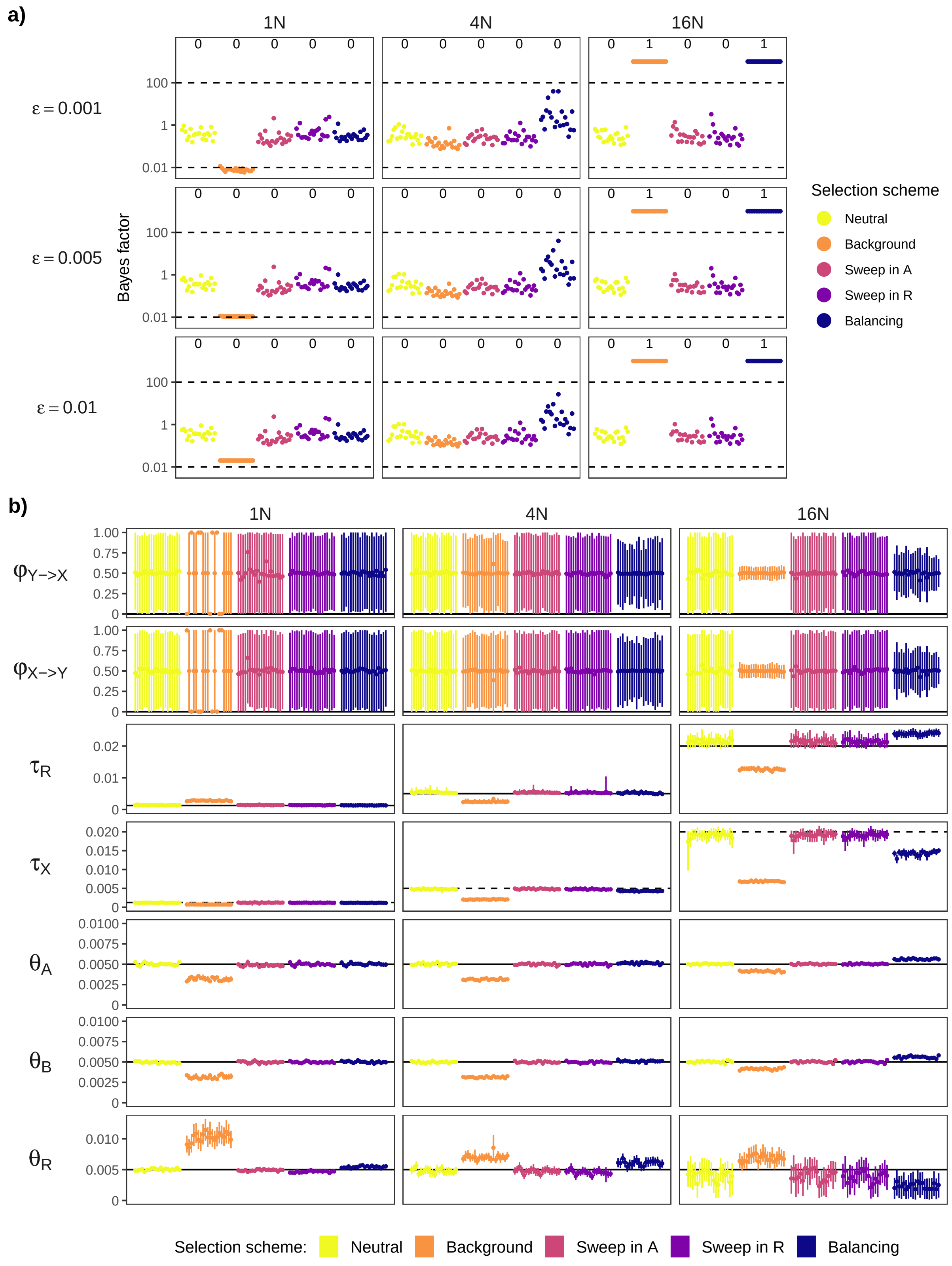


**Fig. S10**: **a)** Bayes factors and false positive rates using the BDI model (fig. 1**b**), assuming $\theta_X = \theta_A$ and $\theta_Y = \theta_B$, for data simulated under different selection schemes with no recombinations. **b)** Parameter estimates for data of panel **a**. See legend to Figure S1.

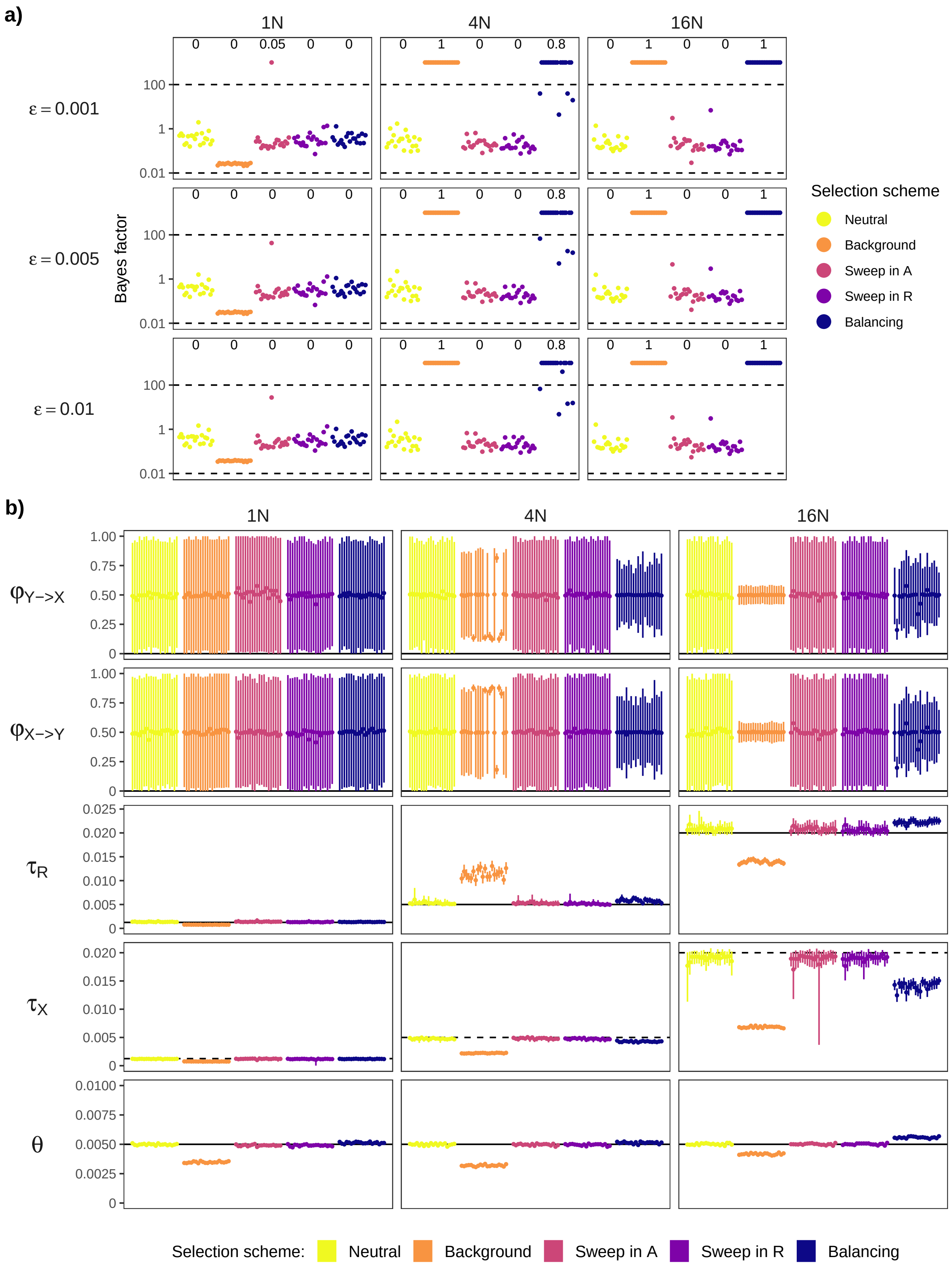


**Fig. S11**: **a)** Bayes factors and false positive rates under the BDI model (fig. 1**b**), assuming all populations have the same size ($\theta$), for data simulated under different selection schemes with no recombinations. **b)** Parameter estimates for data of panel **a**. See legend to Figure S1.

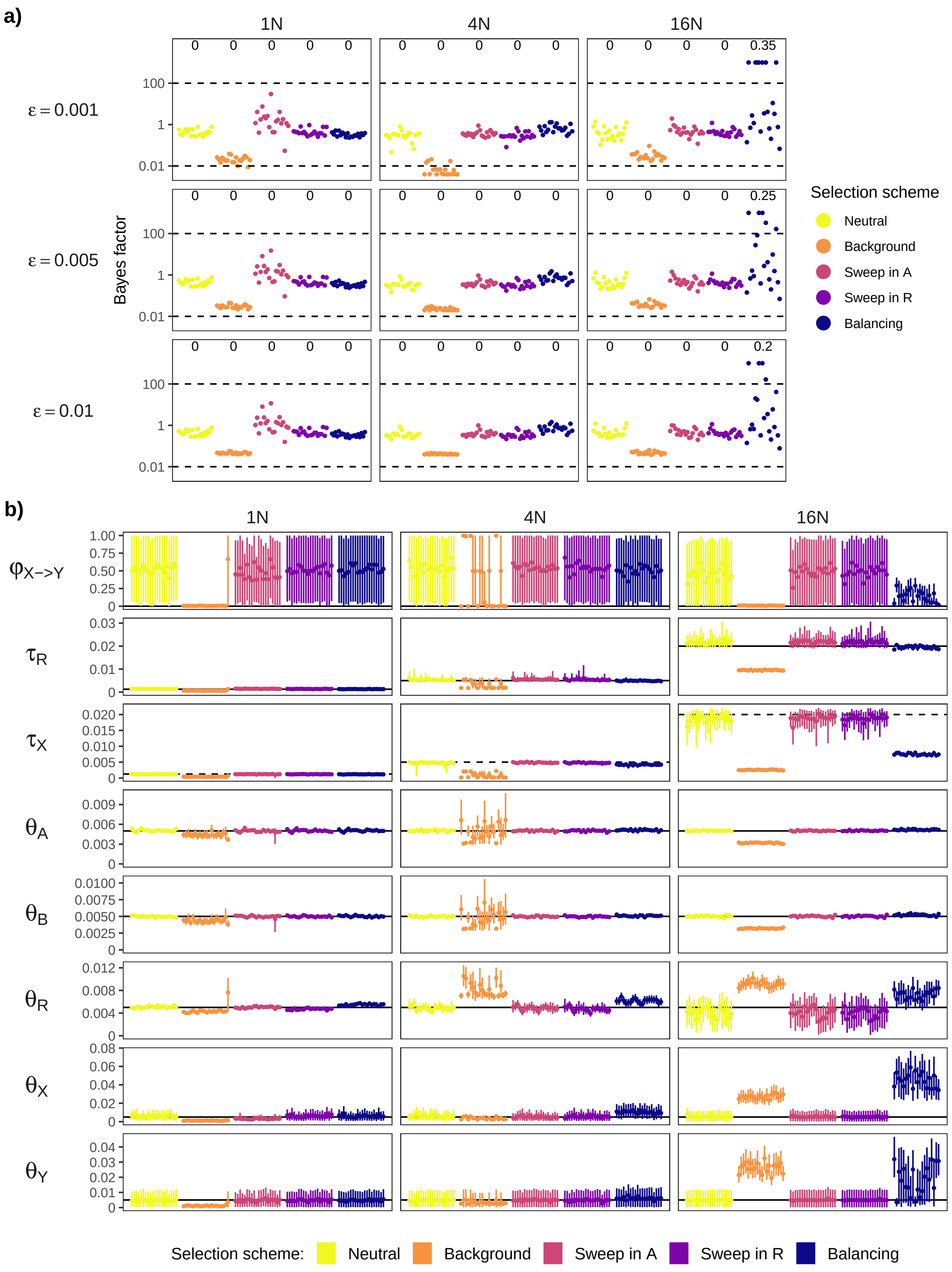


**Fig. S12**: **a)** Bayes factors and false positive rates under the UDI model (fig. 1**c**), for data simulated under different selection schemes with no recombinations. **b)** Parameter estimates for data of panel **a**. See legend to Figure S1.

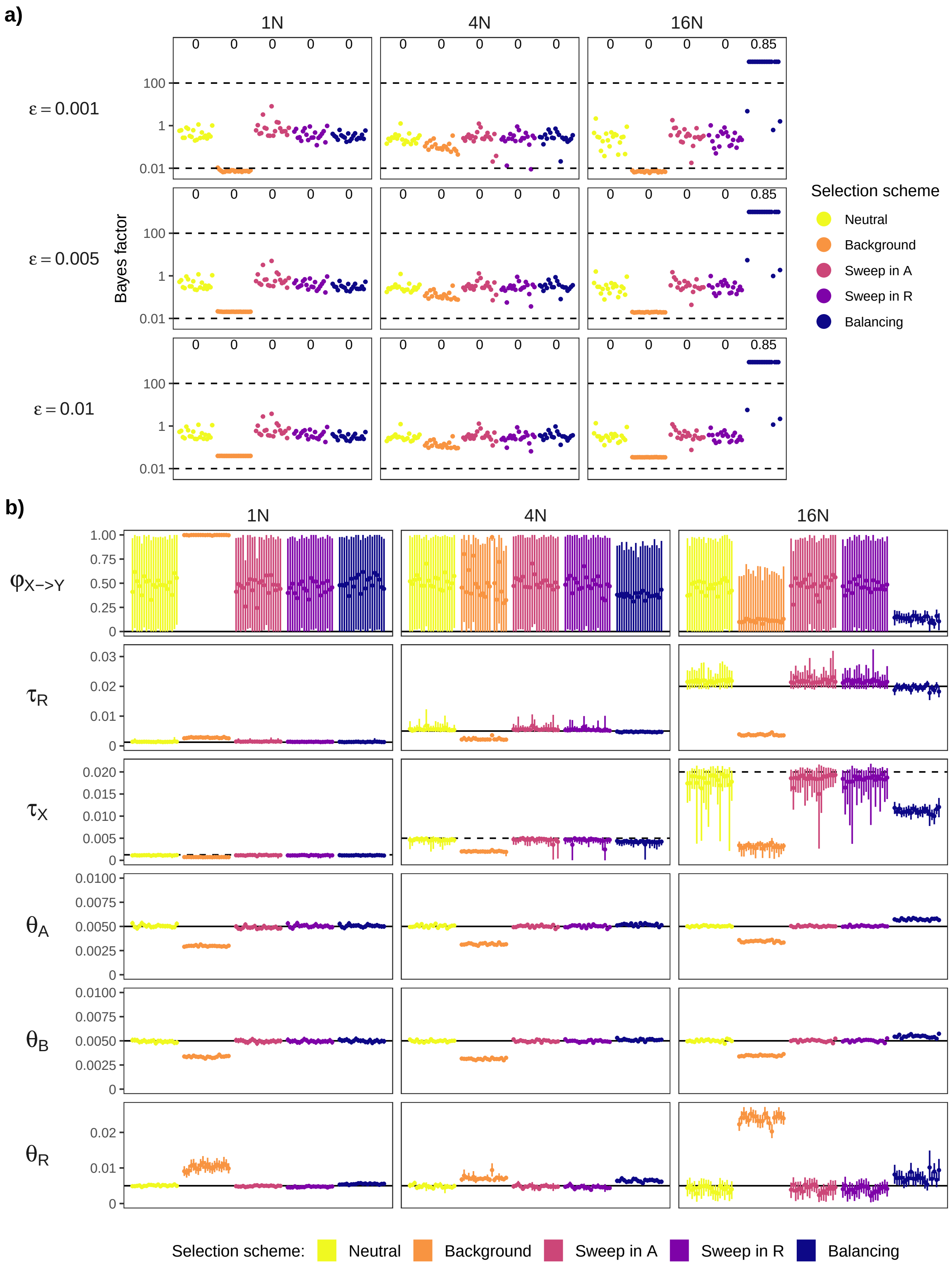


**Fig. S13**: **a)** Bayes factors and false positive rates under the UDI model (fig. 1**c**), assuming $\theta_X = \theta_A$ and $\theta_Y = \theta_B$, for data simulated under different selection schemes with no recombinations. **b)** Parameter estimates for data of panel **a**. See legend to Figure S1.

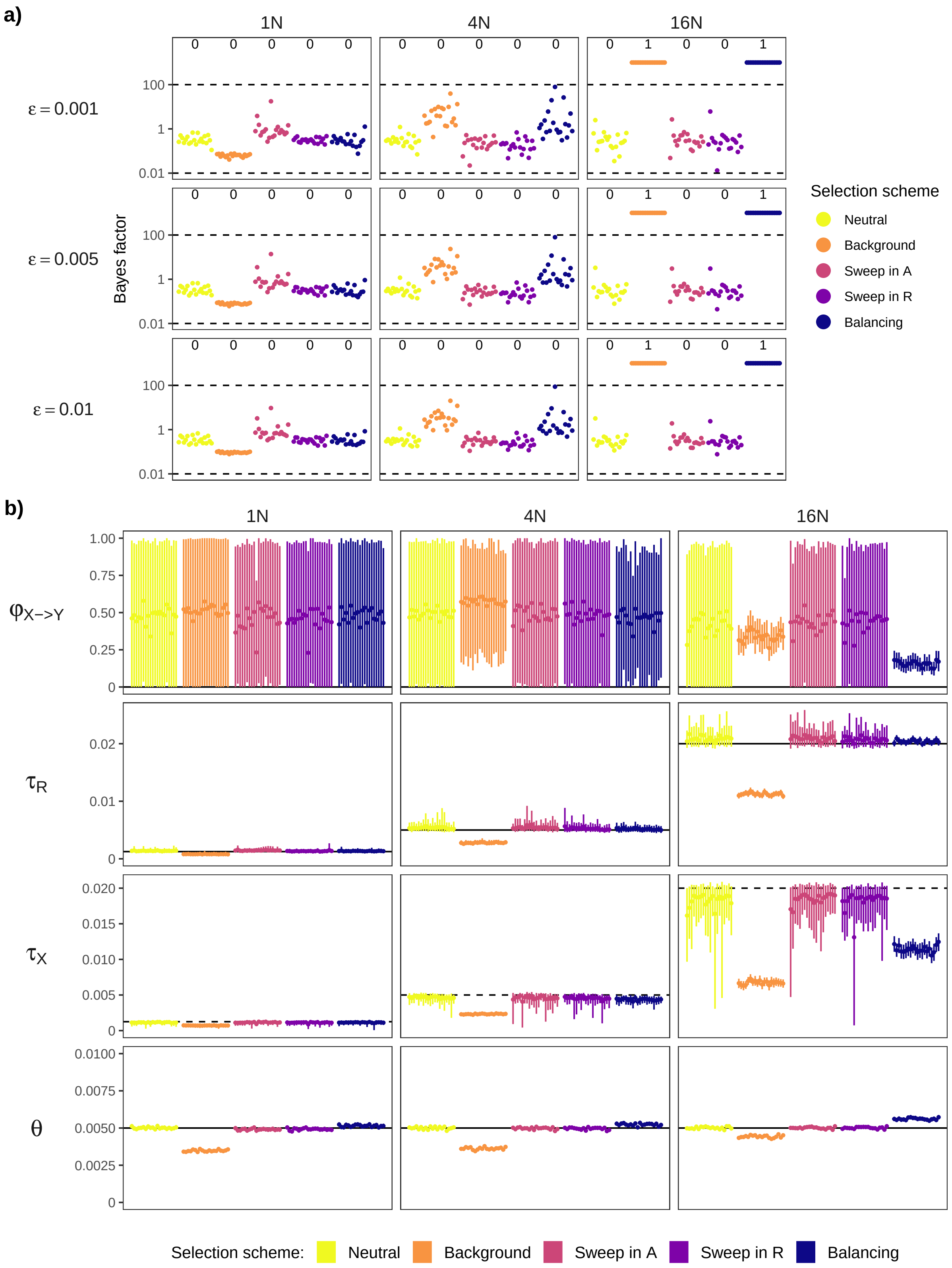


**Fig. S14**: **a)** Bayes factors and false positive rates under the UDI model (fig. 1**c**), assuming all populations have the same size ($\theta$), for data simulated under different selection schemes with no recombinations. **b)** Parameter estimates for data of panel **a**. See legend to Figure S1.

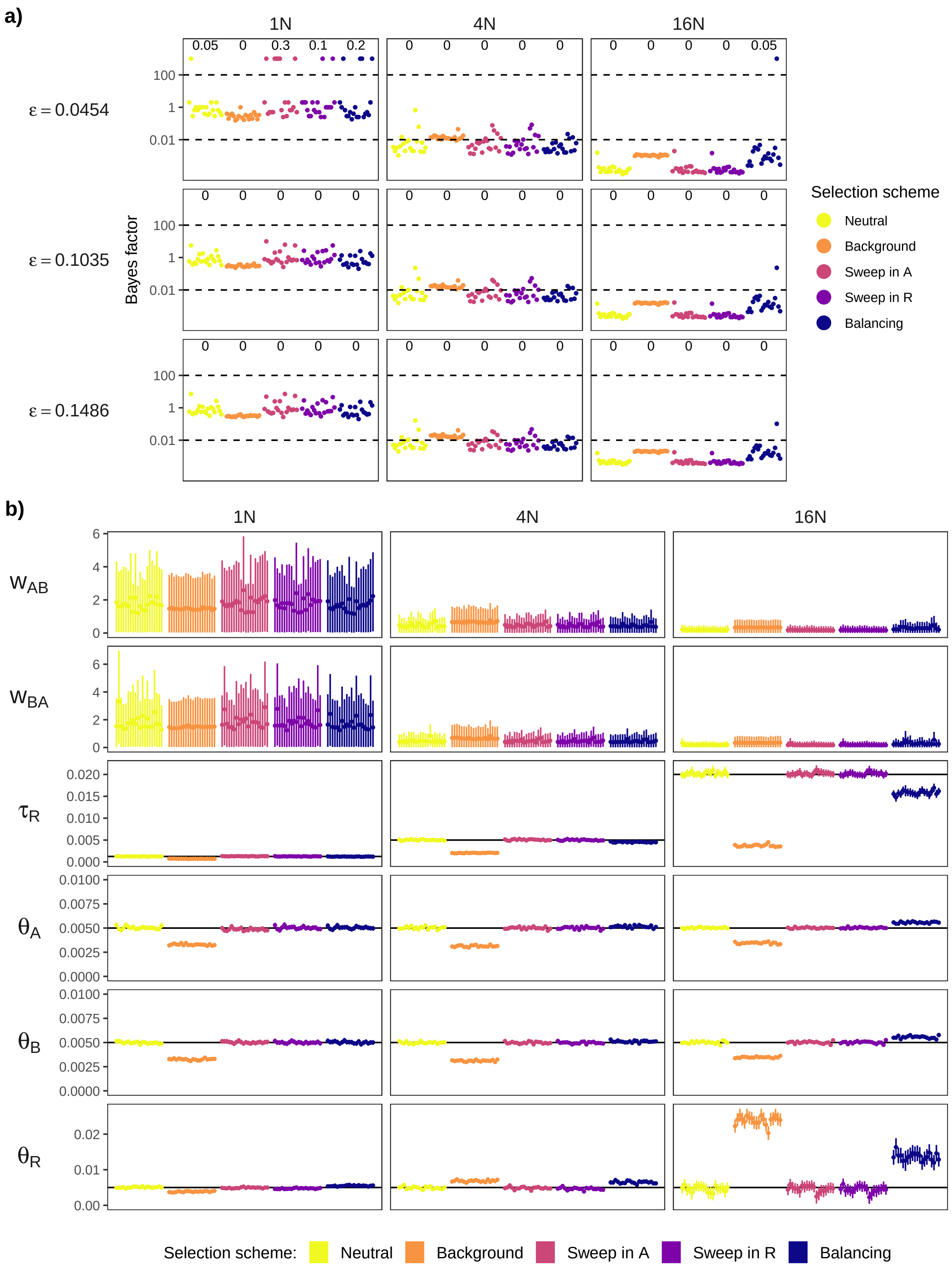


**Fig. S15**: **a)** Bayes factors and false positive rates under the BDM model (fig. 1**d**), for data simulated under different selection schemes with no recombinations. **b)** Parameter estimates for data of panel **a**. See legend to Figure S1.

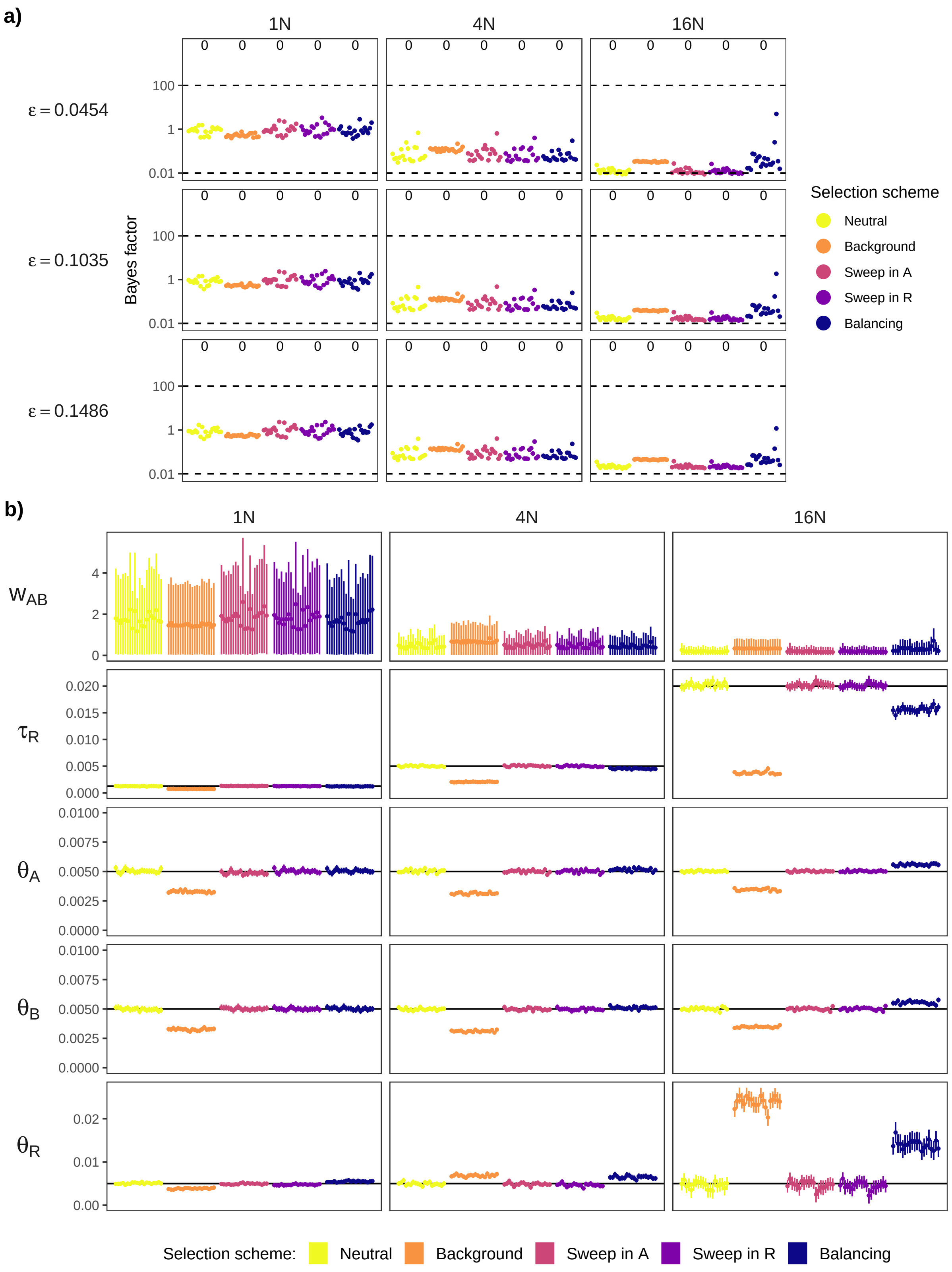


**Fig. S16**: **a)** Bayes factors and false positive rates under the UDM model (fig. 1**e**), for data simulated under different selection schemes with no recombinations. **b)** Parameter estimates for data of panel **a**. See legend to Figure S1.

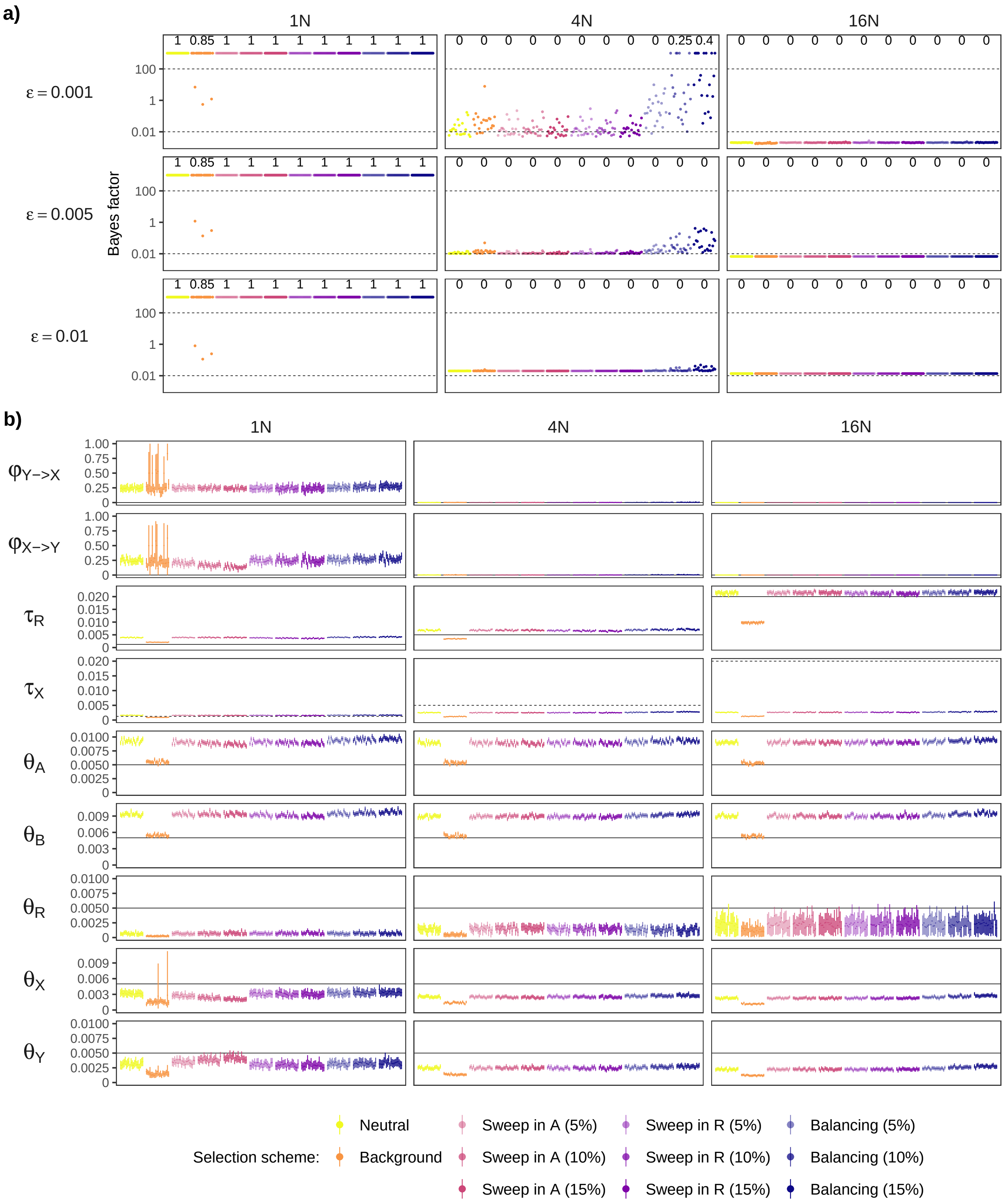


**Fig. S17**: **a)** Bayes factors and false positive rates under the BDI model (fig. 1**b**) for data of Smith and Hahn (2024) simulated under different selection schemes with recombination rate $\rho = 0.025$. For selective sweeps and balancing selection, three proportions of loci under selection were considered: 5%, 10%, and 15%. **b)** Parameter estimates for data of panel **a**. See legend to Figure S1.

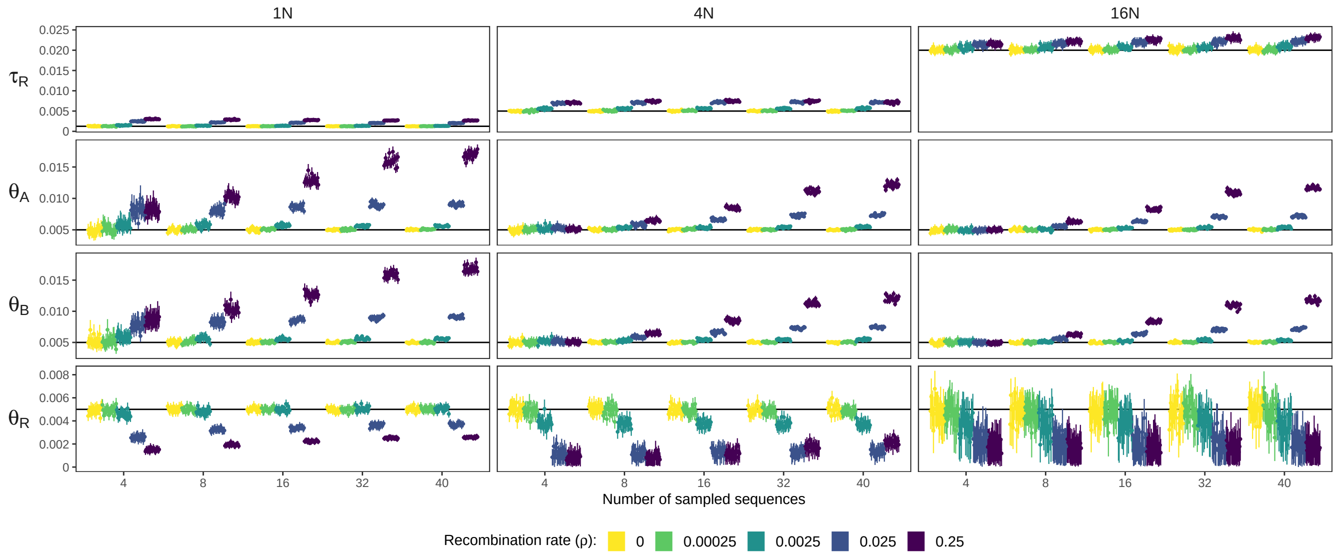


**Fig. S18**: Posterior means and 95% HPD CIs of parameters in the MSC model (fig. 1**a**) in datasets of different numbers of sequences simulated under neutral evolution at different recombination rates. Bayes factors for the test of introgression under the BDI model for those data are shown in figure 3.

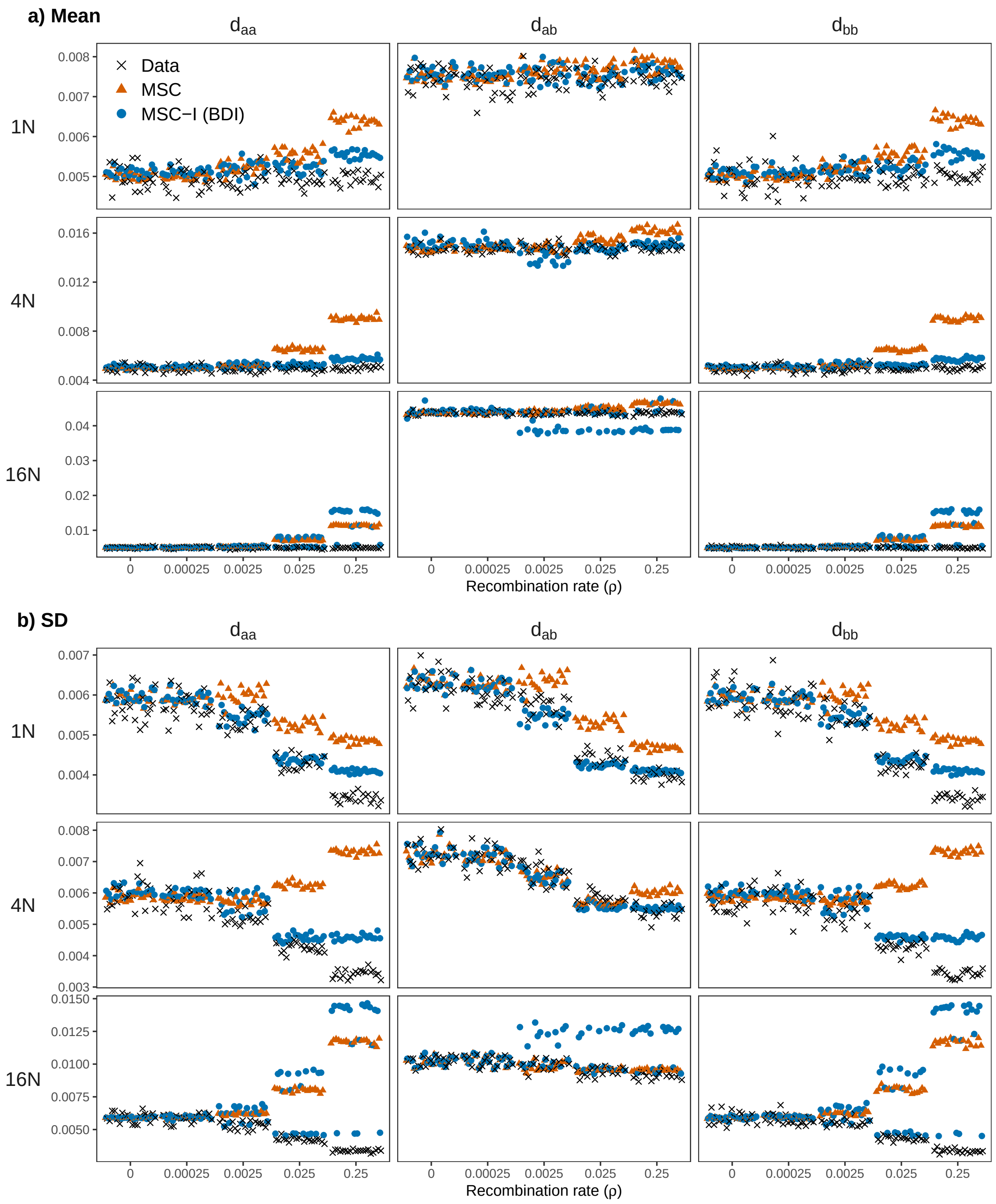


**Fig. S19**: (**a**) Means and (**b**) standard deviations in the observed and predicted pairwise sequence distances at different recombination rates for neutral evolution. Observed distances ($d_{aa}$ and $d_{bb}$ within species and $d_{ab}$ between species) are calculated by using a pair of sequences per locus. Predicted distances, under the MSC model with no gene flow and the MSC-I model with bidirectional introgression (BDI; fig. 1**b**), are calculated using the posterior means of parameters obtained from the BPP analyses of the data (fig. S1**b**); see Appendix B.

**Table S1**: Summary of simulation scenarios and analysis models

| Model | $T$ | $s$ | $L$ | $n$ | $\rho = 4Nr$ | Selection scheme | Simulation software | Analysis model | Results |
|---|---|---|---|---|---|---|---|---|---|
| fig. 1a | $N, 4N, 16N$ | 20 | 500 | 500 | 0, 0.00025, 0.00025, 0.0025, 0.025, 0.25 | neutral | SLiM + pyslim + tskit | BDI (fig. 1b), unconstrained $\theta$ | figs. 2a, S1, S19 |
| | | | | | | | | BDI (fig. 1b), linked $\theta$ | fig. S2 |
| | | | | | | | | BDI (fig. 1b), same $\theta$ | fig. S3 |
| | | | | | | | | UDI (fig. 1c), unconstrained $\theta$ | fig. S4 |
| | | | | | | | | UDI (fig. 1c), linked $\theta$ | fig. S5 |
| | | | | | | | | UDI (fig. 1c), same $\theta$ | fig. S6 |
| | | | | | | | | BDM (fig. 1d) | fig. S7 |
| | | | | | | | | UDM (fig. 1e) | figs. 7, S8 |
| | | | | | 0 | background, sweep in A, sweep in R, balancing | SLiM + pyslim + tskit | BDI (fig. 1b), unconstrained $\theta$ | figs. 2b, S9 |
| | | | | | | | | BDI (fig. 1b), linked $\theta$ | fig. S10 |
| | | | | | | | | BDI (fig. 1b), same $\theta$ | fig. S11 |
| | | | | | | | | UDI (fig. 1c), unconstrained $\theta$ | fig. S12 |
| | | | | | | | | UDI (fig. 1c), linked $\theta$ | fig. S13 |
| | | | | | | | | UDI (fig. 1c), same $\theta$ | fig. S14 |
| | | | | | | | | BDM (fig. 1d) | fig. S15 |
| | | | | | | | | UDM (fig. 1e) | fig. S16 |
| fig. 1a | $N, 4N, 16N$ | 2, 4, 8, 16, 20 | 500 | 500 | 0, 0.00025, 0.00025, 0.0025, 0.025, 0.25 | neutral | ms + SeqGen | BDI (fig. 1b), unconstrained $\theta$ | figs. 3, S18 |
| | | | | | | | | MSC (fig. 1a), for s = 2 only | fig. 6 |
| fig. 1a | $N, 4N, 16N$ | 20 | 500 | 100 | 0, 0.00025, 0.00025, 0.0025, 0.025, 0.25 | neutral | SLiM + pyslim + tskit | BDI (fig. 1b), unconstrained $\theta$ | fig. 4a |
| | | | | | 0 | background, sweep in $A$, sweep in $R$, balancing | SLiM + pyslim + tskit | BDI (fig. 1b), unconstrained $\theta$ | fig. 4b |
| fig. 1a | $N, 4N, 16N$ | 20 | 500 | 500 | 0.025 | neutral, background, sweep in A, sweep in R, balancing | SLiM + pyslim + tskit (data from Smith & Hahn, 2024) | BDI (fig. 1b), unconstrained $\theta$ | fig. S17 |
| fig. 5a | $N, 4N$ | 20 from $A, B, C$; 1 from $O$ | 500 | 500 | 0, 0.00025, 0.00025, 0.0025, 0.025, 0.25 | neutral | ms + SeqGen | fig. 5b | fig. 5c |

Note.— $T$ is divergence time (in generations), $s$ is the number of sequences per species, $L$ is the number of loci, $n$ is number of sites per locus (sequence length), $\rho = 4Nr$ is the population recombination rate. The number of replicates is 20 for each setting.
